# Supplementary figures and images for: Viewpoint Planning for Range Sensors Using Feature Cluster Constrained Spaces for Robot Vision Systems
Source: Sensors (Basel). 2023 Sep 18;23(18):7964. doi: 10.3390/s23187964 (PMC10537344; doi:10.3390/s23187964)

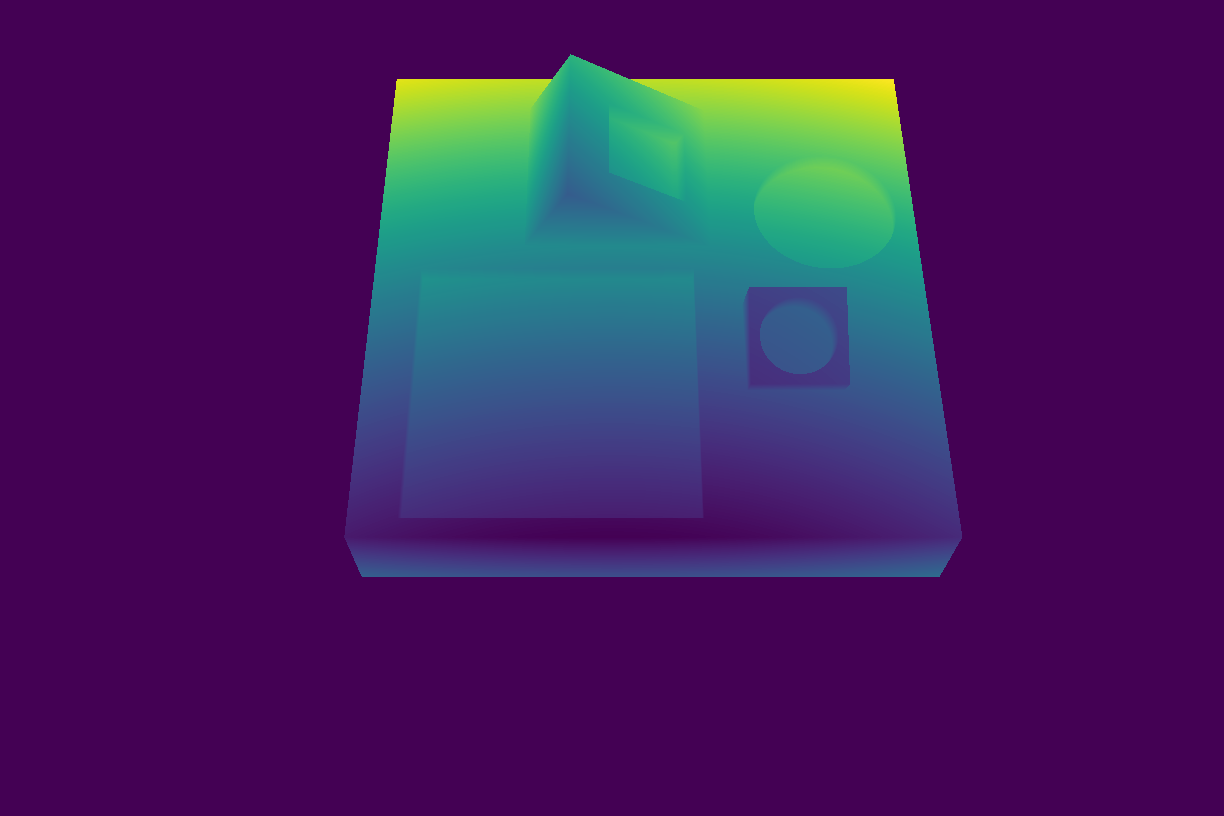

Supplement: Supplementary file 1 [file sensors-23-07964-s001.zip › Academic Example Synthetic/6_GC-Spaces/G1/render/center_depth_image_gcs_G1_s1.png]

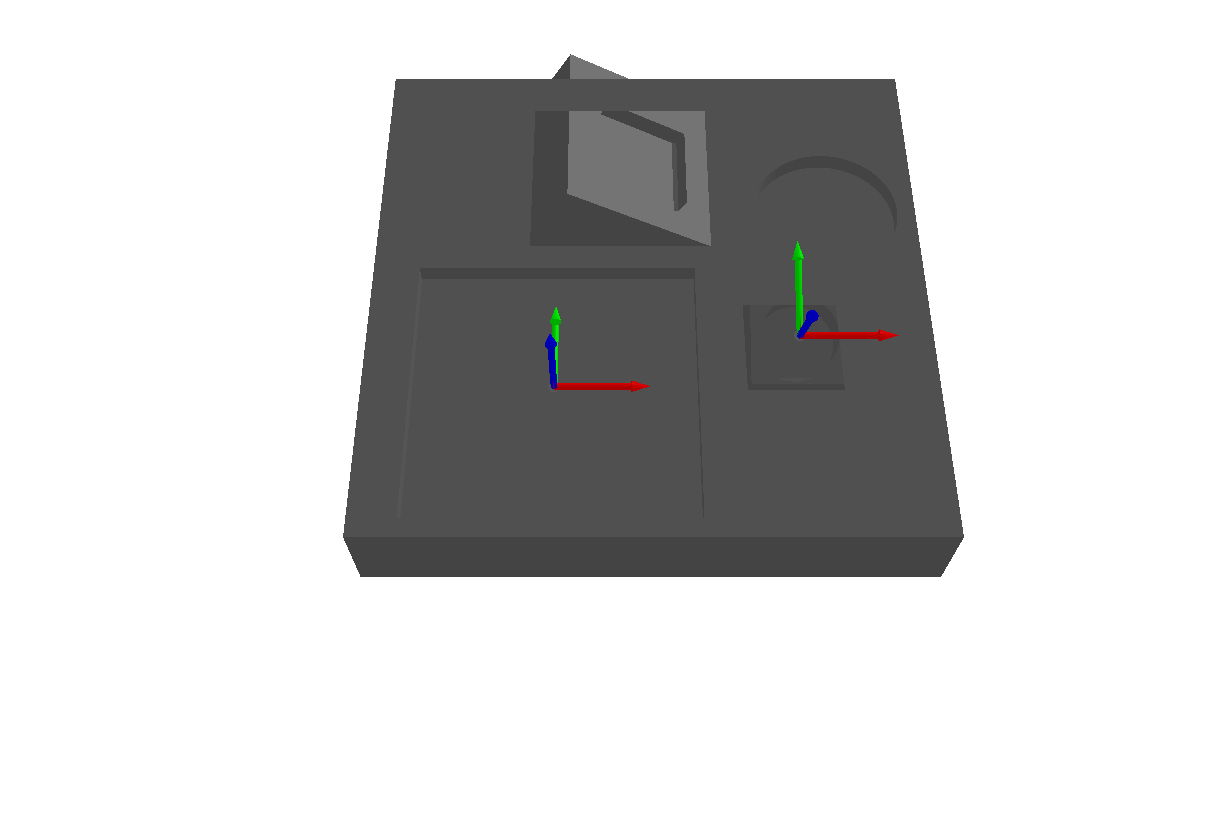

Supplement: Supplementary file 1 [file sensors-23-07964-s001.zip › Academic Example Synthetic/6_GC-Spaces/G1/render/center_render_gcs_G1_s1.png]

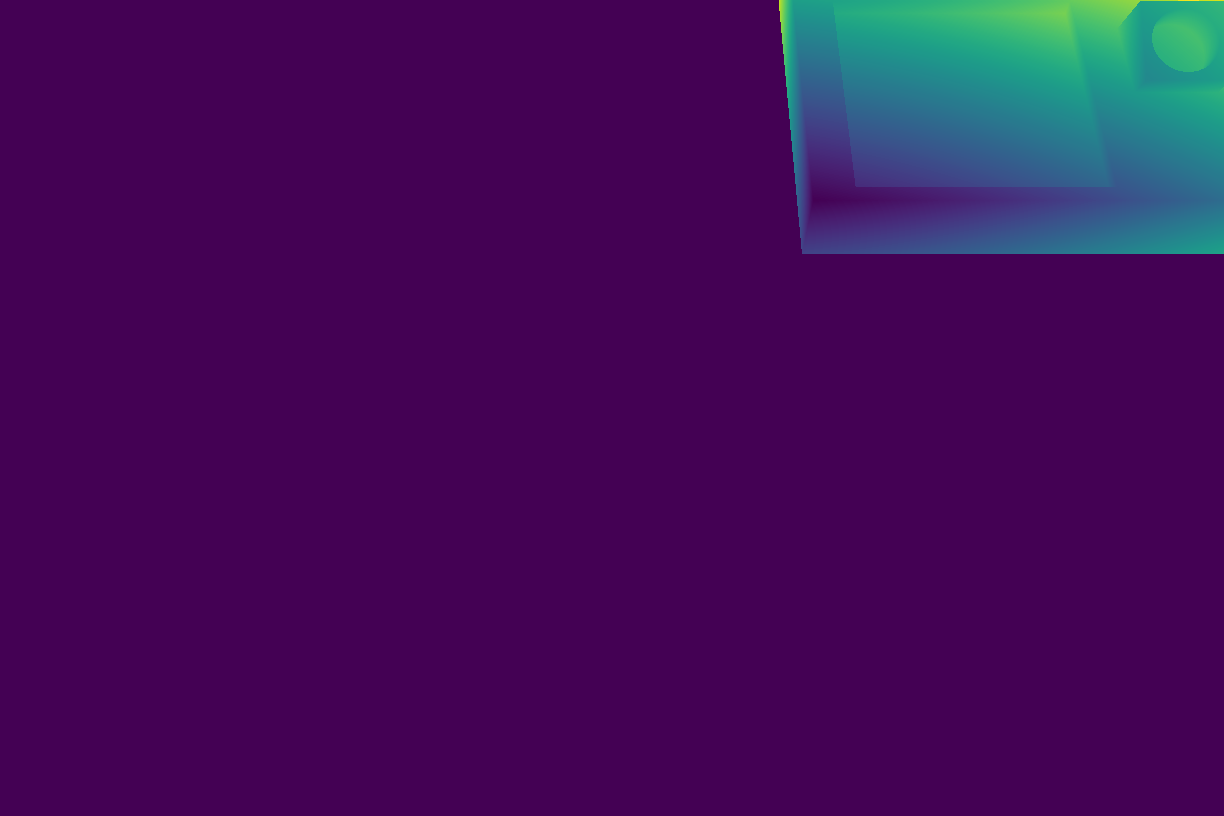

Supplement: Supplementary file 1 [file sensors-23-07964-s001.zip › Academic Example Synthetic/6_GC-Spaces/G1/render/vx0_depth_image_gcs_G1_s1.png]

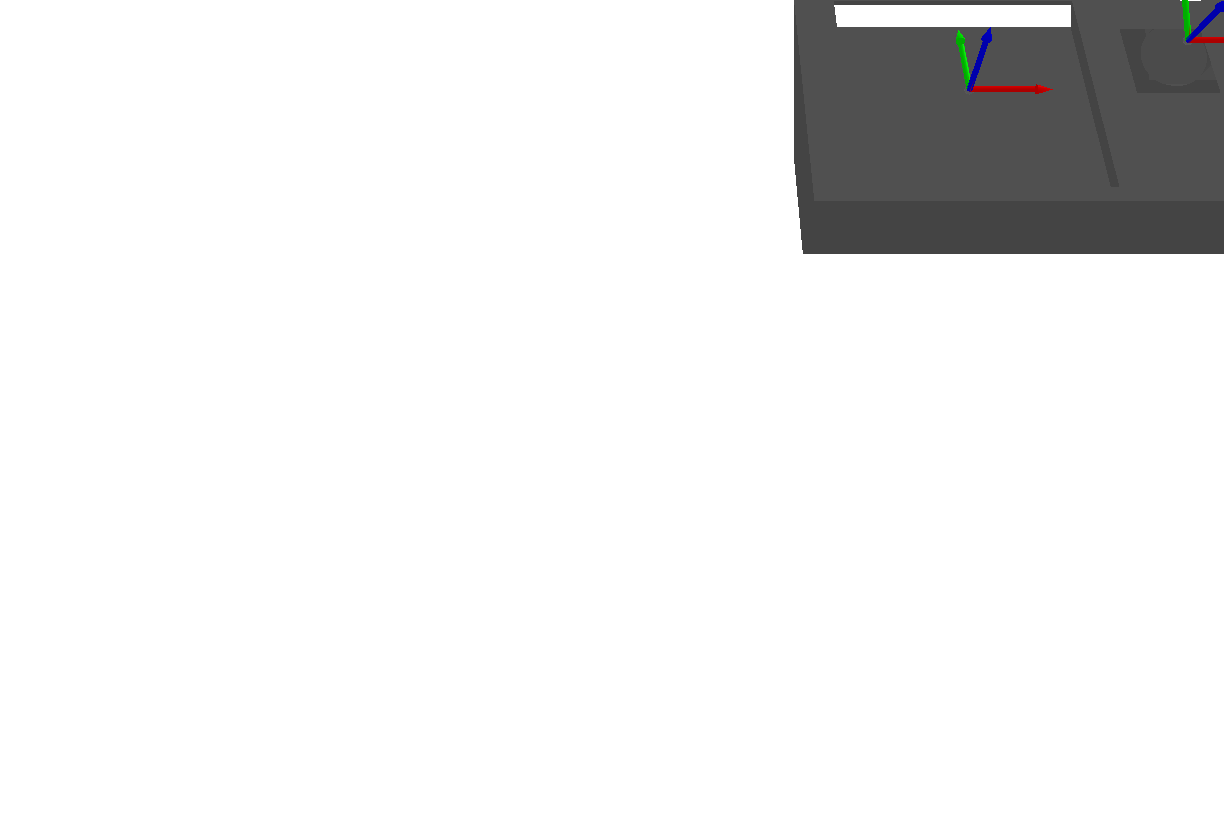

Supplement: Supplementary file 1 [file sensors-23-07964-s001.zip › Academic Example Synthetic/6_GC-Spaces/G1/render/vx0_render_gcs_G1_s1.png]

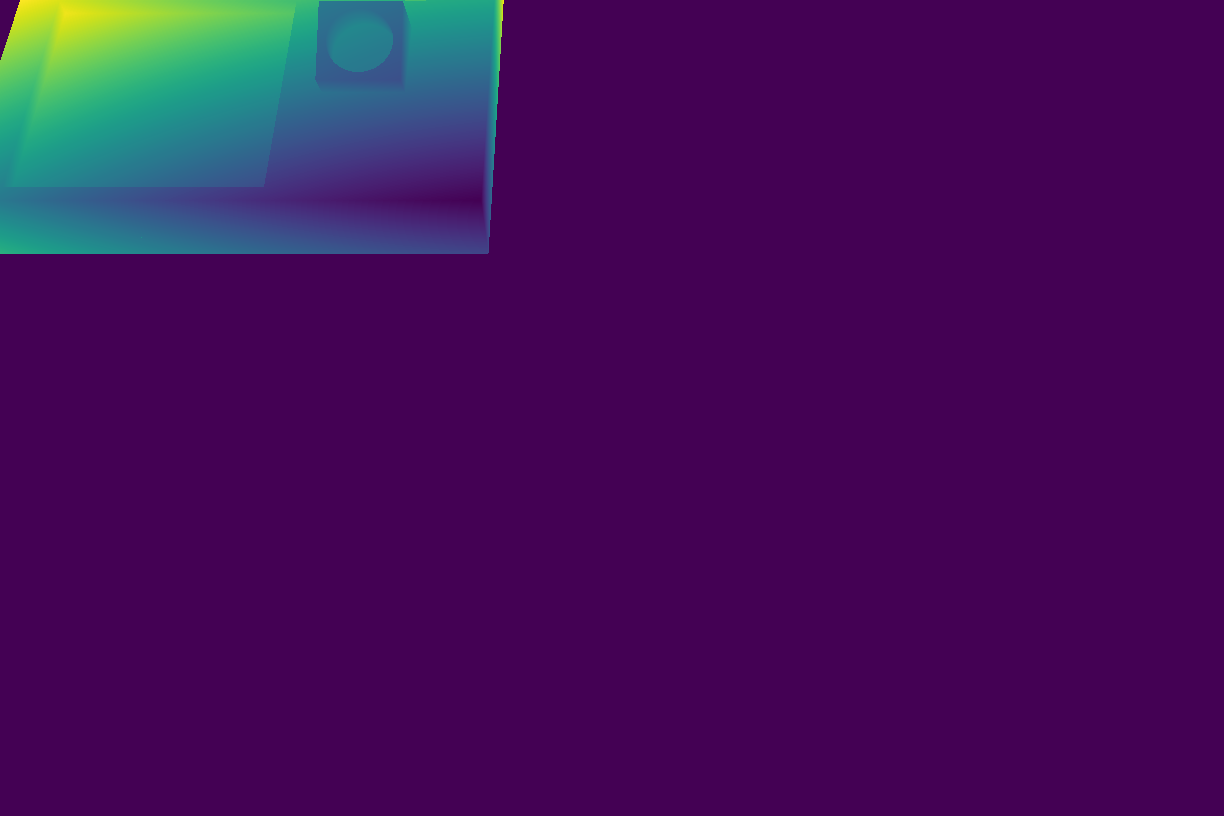

Supplement: Supplementary file 1 [file sensors-23-07964-s001.zip › Academic Example Synthetic/6_GC-Spaces/G1/render/vx1_depth_image_gcs_G1_s1.png]

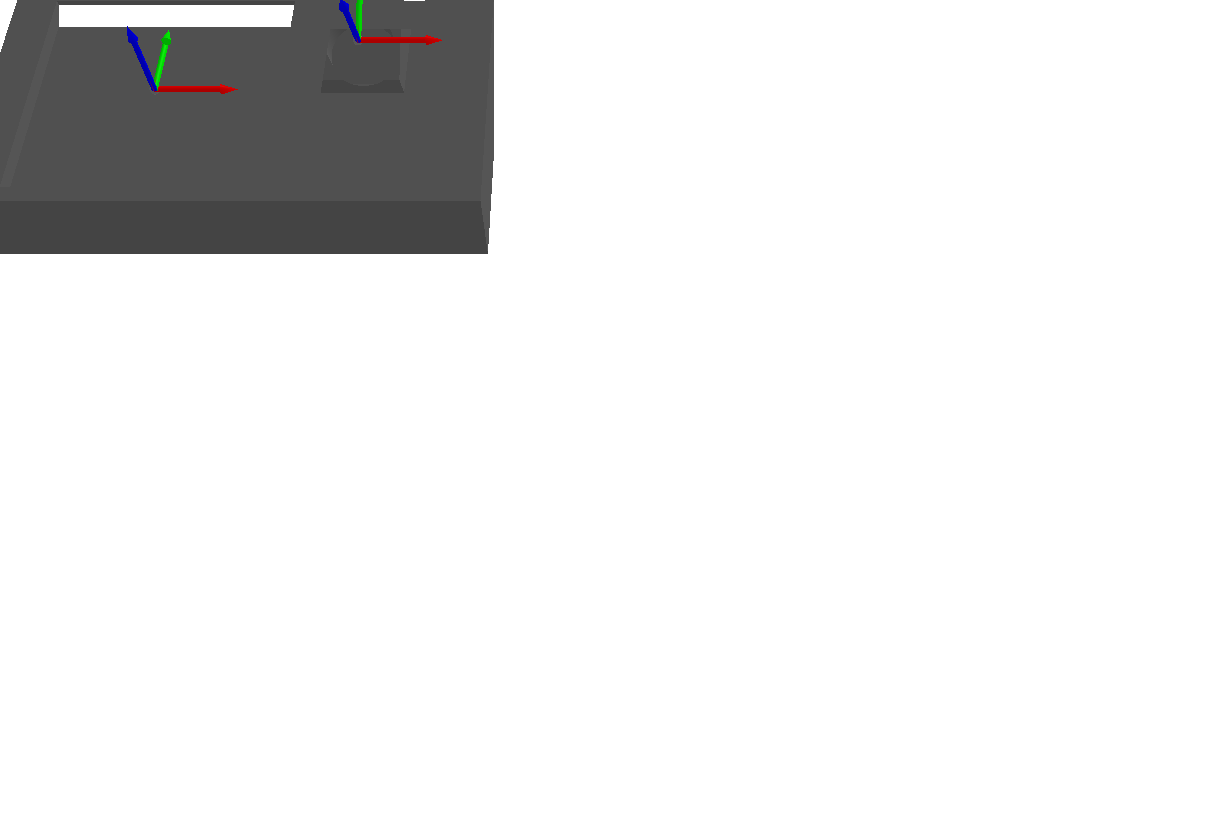

Supplement: Supplementary file 1 [file sensors-23-07964-s001.zip › Academic Example Synthetic/6_GC-Spaces/G1/render/vx1_render_gcs_G1_s1.png]

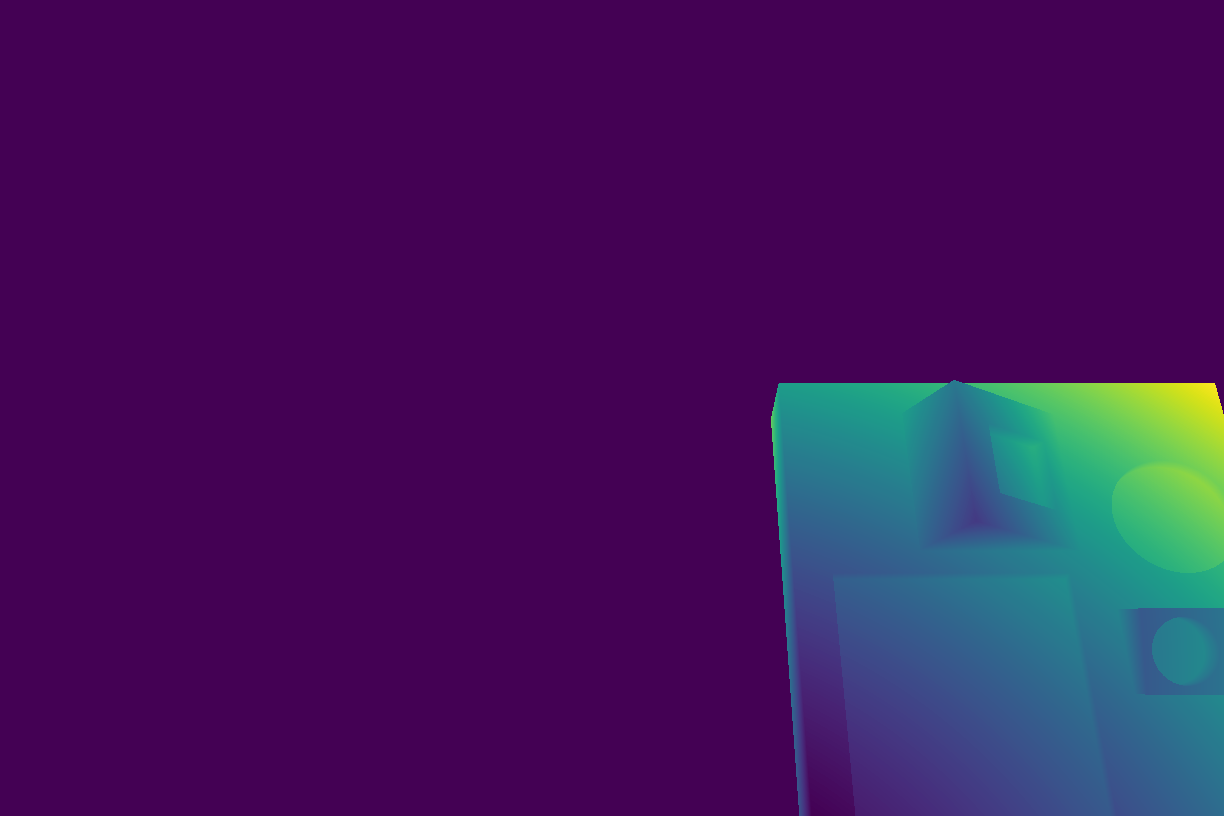

Supplement: Supplementary file 1 [file sensors-23-07964-s001.zip › Academic Example Synthetic/6_GC-Spaces/G1/render/vx2_depth_image_gcs_G1_s1.png]

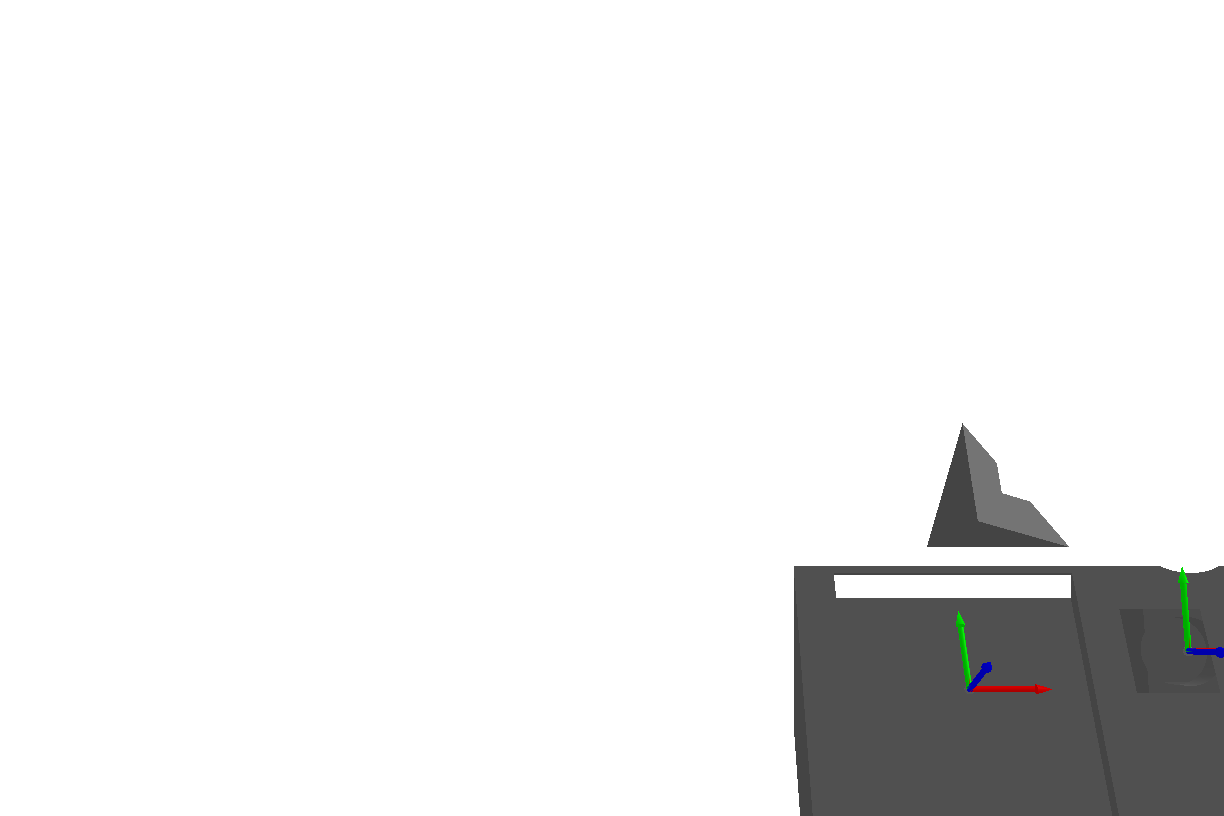

Supplement: Supplementary file 1 [file sensors-23-07964-s001.zip › Academic Example Synthetic/6_GC-Spaces/G1/render/vx2_render_gcs_G1_s1.png]

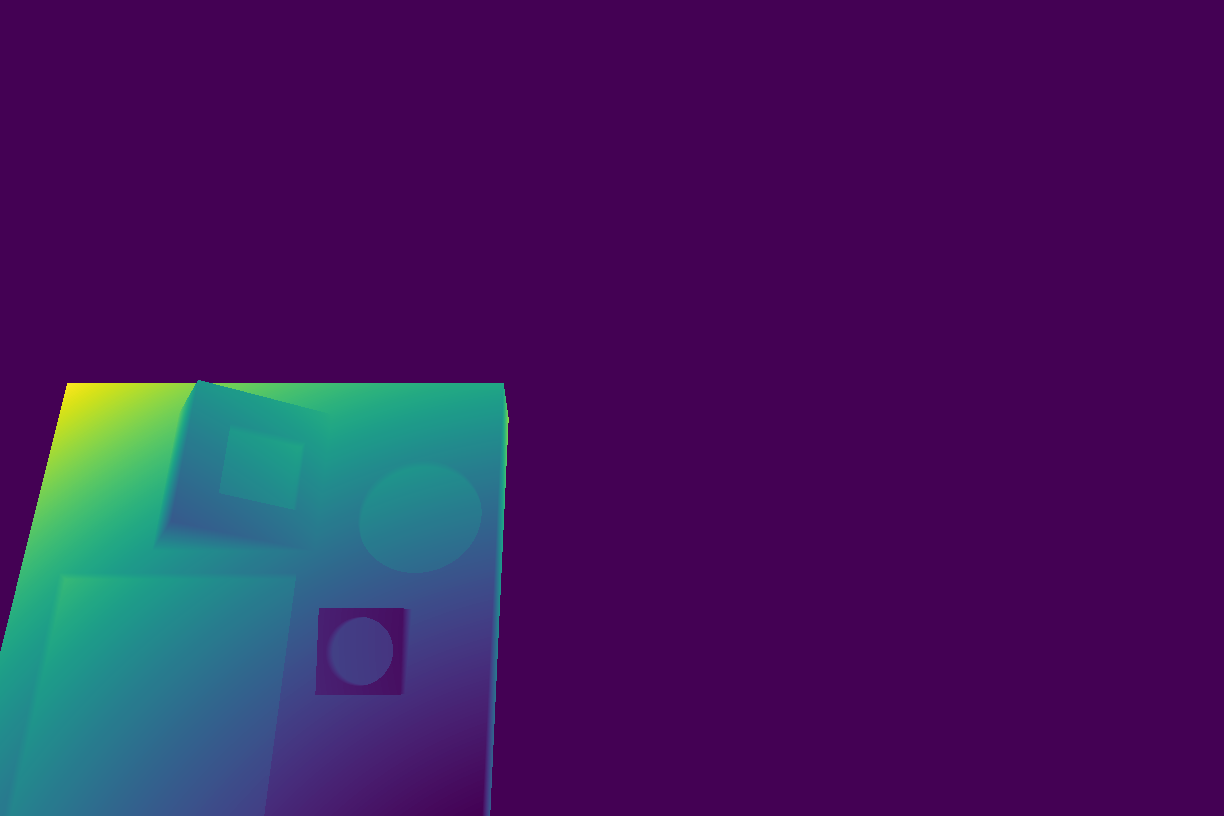

Supplement: Supplementary file 1 [file sensors-23-07964-s001.zip › Academic Example Synthetic/6_GC-Spaces/G1/render/vx3_depth_image_gcs_G1_s1.png]

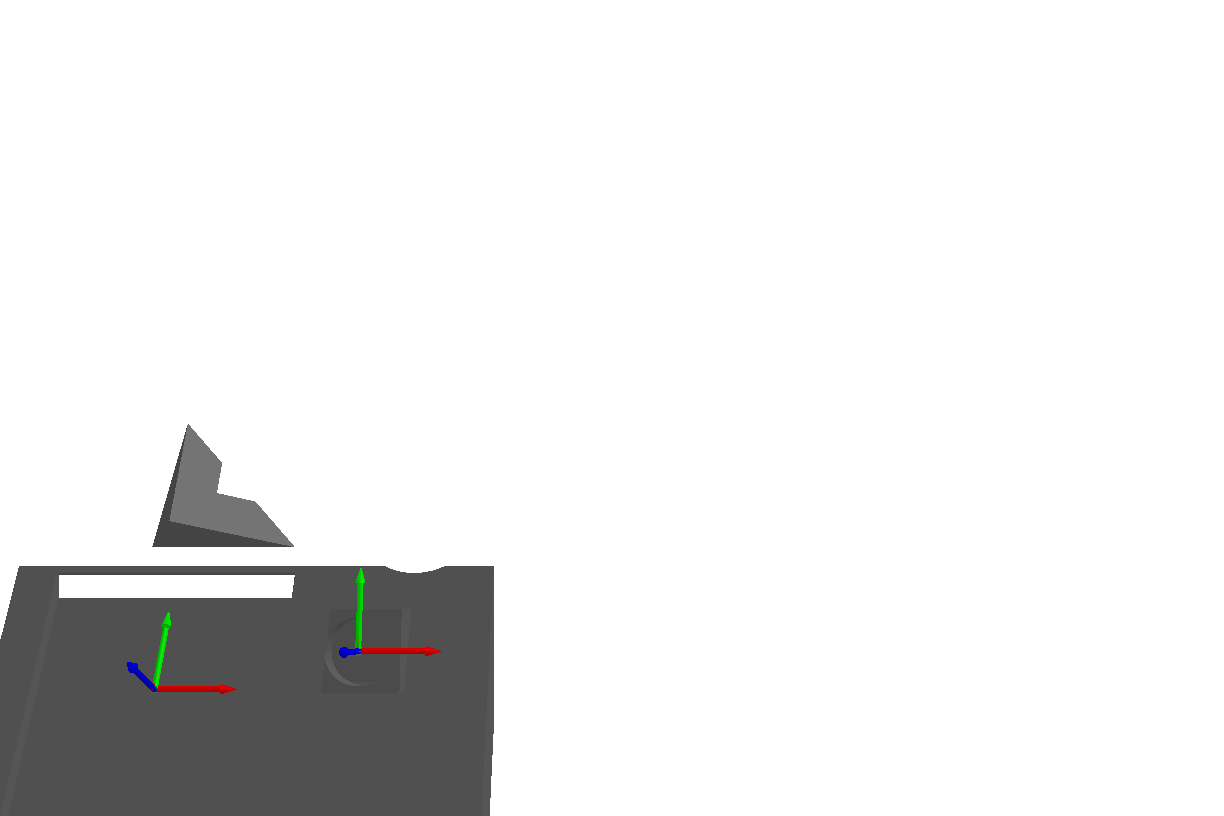

Supplement: Supplementary file 1 [file sensors-23-07964-s001.zip › Academic Example Synthetic/6_GC-Spaces/G1/render/vx3_render_gcs_G1_s1.png]

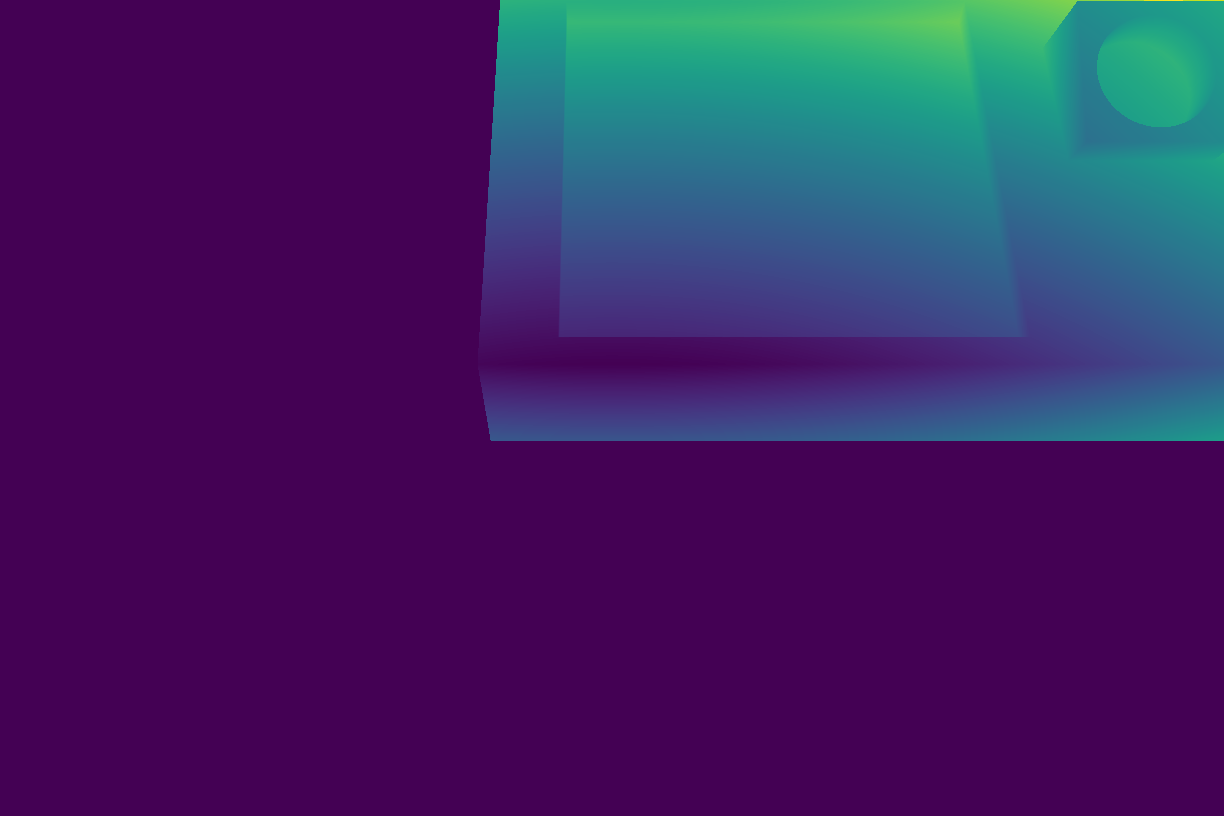

Supplement: Supplementary file 1 [file sensors-23-07964-s001.zip › Academic Example Synthetic/6_GC-Spaces/G1/render/vx4_depth_image_gcs_G1_s1.png]

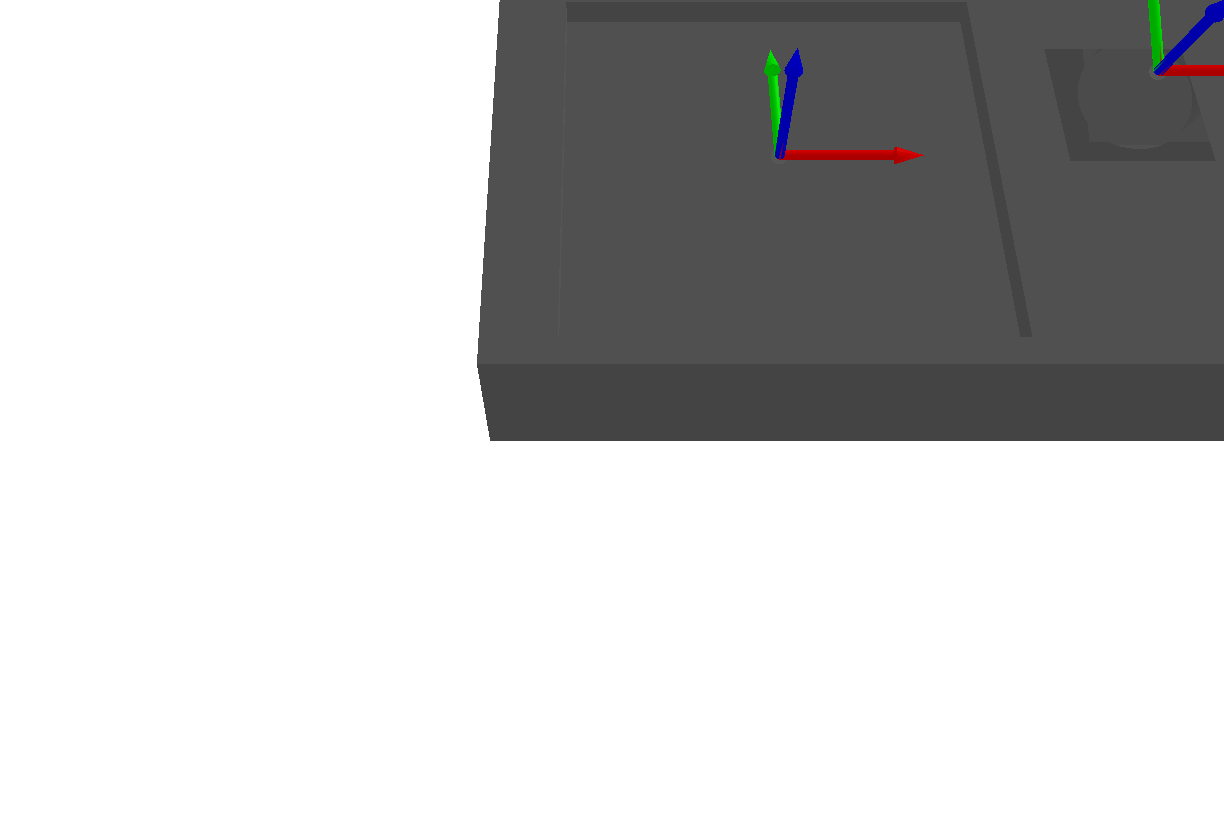

Supplement: Supplementary file 1 [file sensors-23-07964-s001.zip › Academic Example Synthetic/6_GC-Spaces/G1/render/vx4_render_gcs_G1_s1.png]

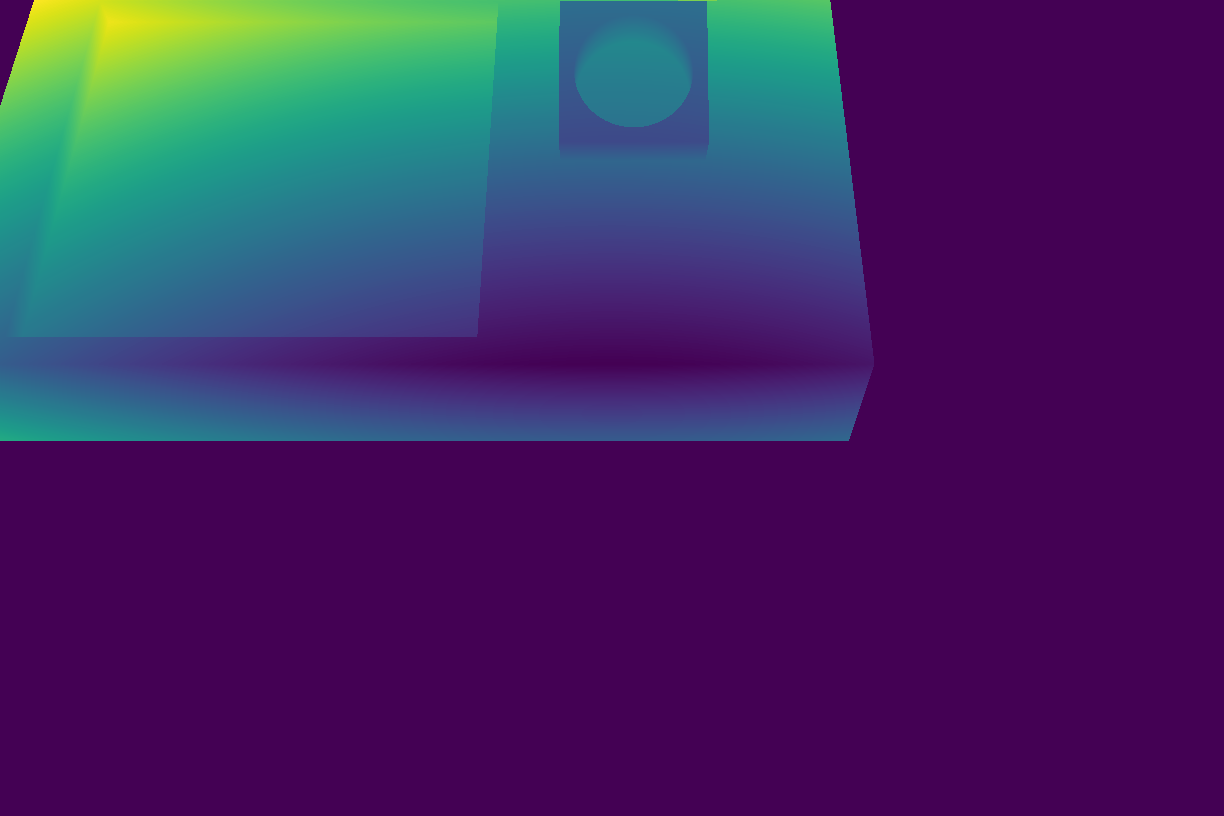

Supplement: Supplementary file 1 [file sensors-23-07964-s001.zip › Academic Example Synthetic/6_GC-Spaces/G1/render/vx5_depth_image_gcs_G1_s1.png]

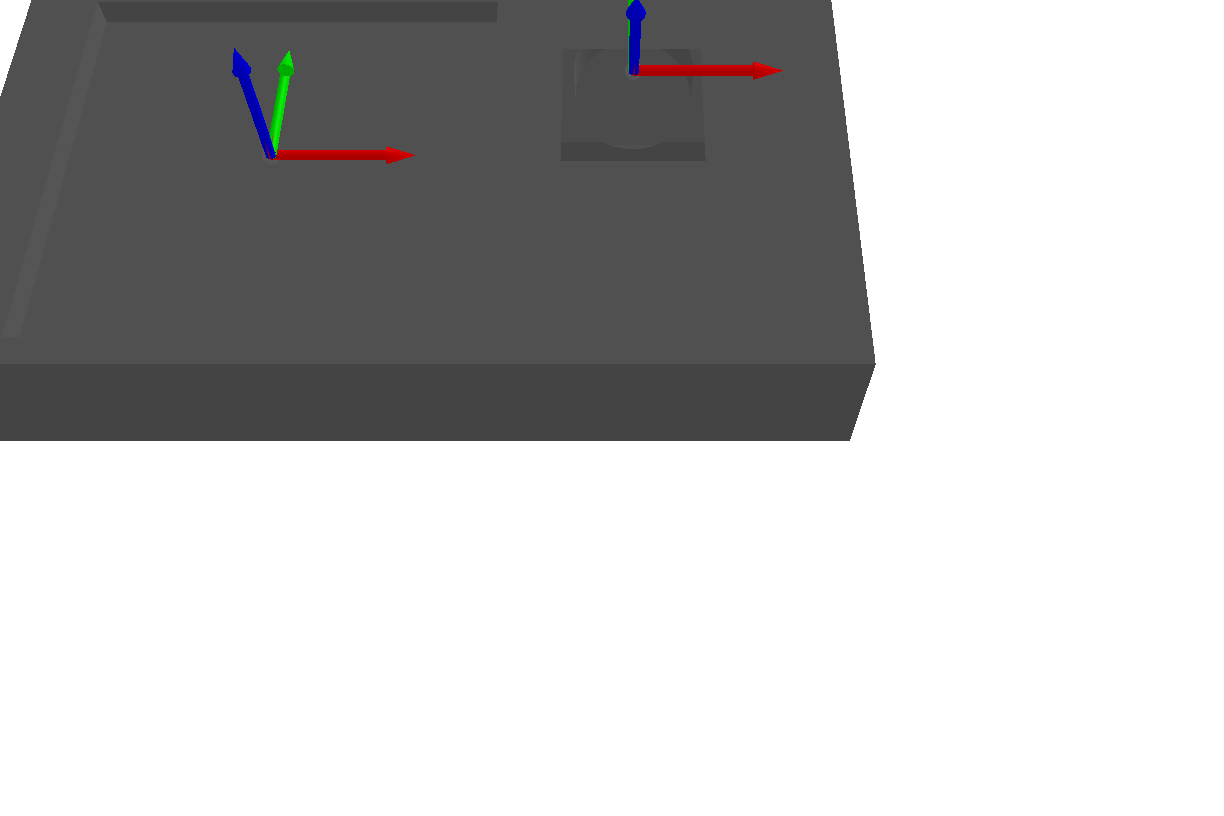

Supplement: Supplementary file 1 [file sensors-23-07964-s001.zip › Academic Example Synthetic/6_GC-Spaces/G1/render/vx5_render_gcs_G1_s1.png]

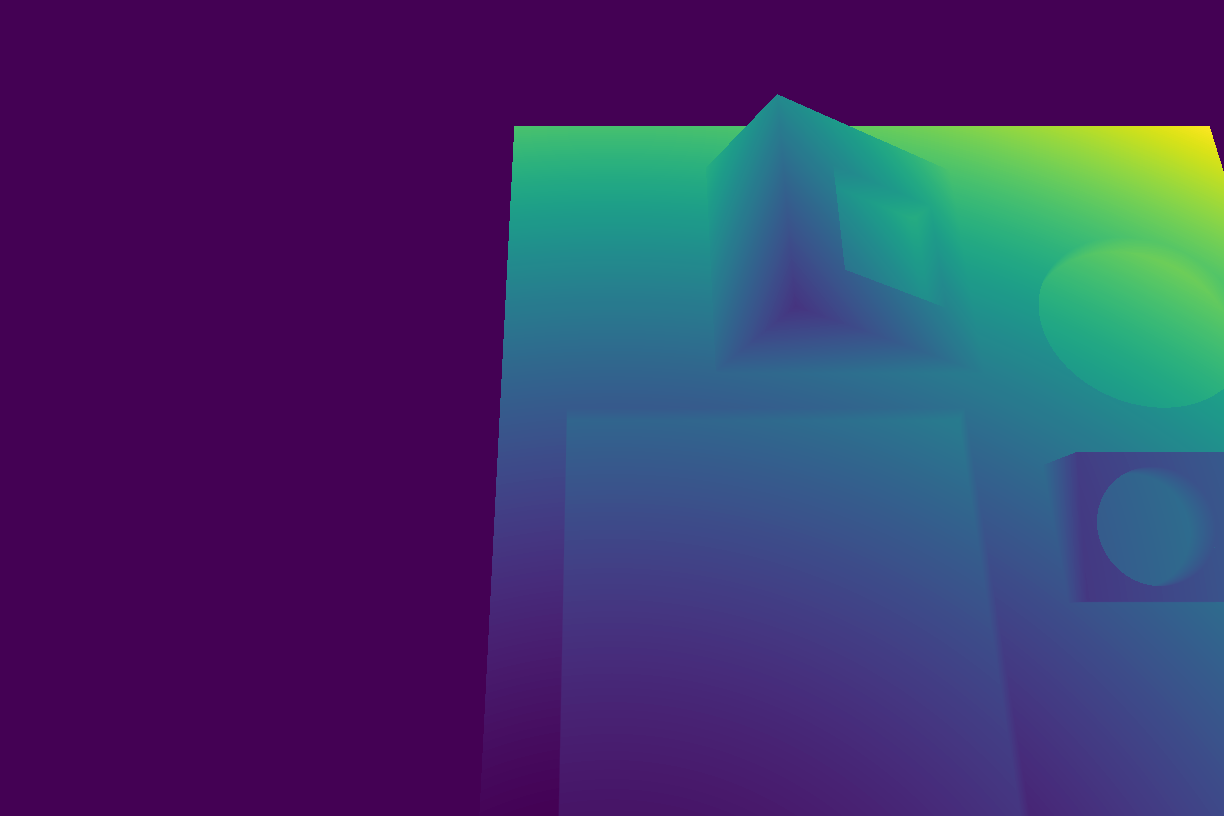

Supplement: Supplementary file 1 [file sensors-23-07964-s001.zip › Academic Example Synthetic/6_GC-Spaces/G1/render/vx6_depth_image_gcs_G1_s1.png]

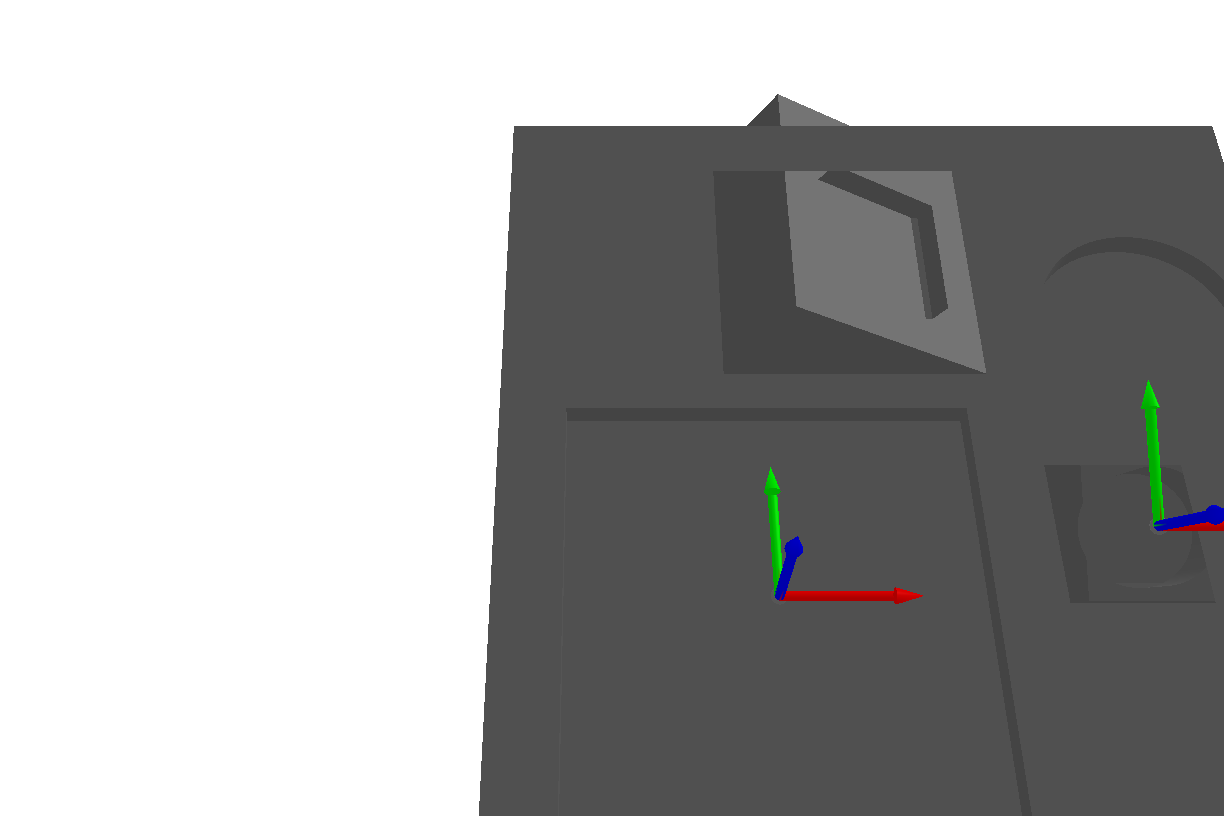

Supplement: Supplementary file 1 [file sensors-23-07964-s001.zip › Academic Example Synthetic/6_GC-Spaces/G1/render/vx6_render_gcs_G1_s1.png]

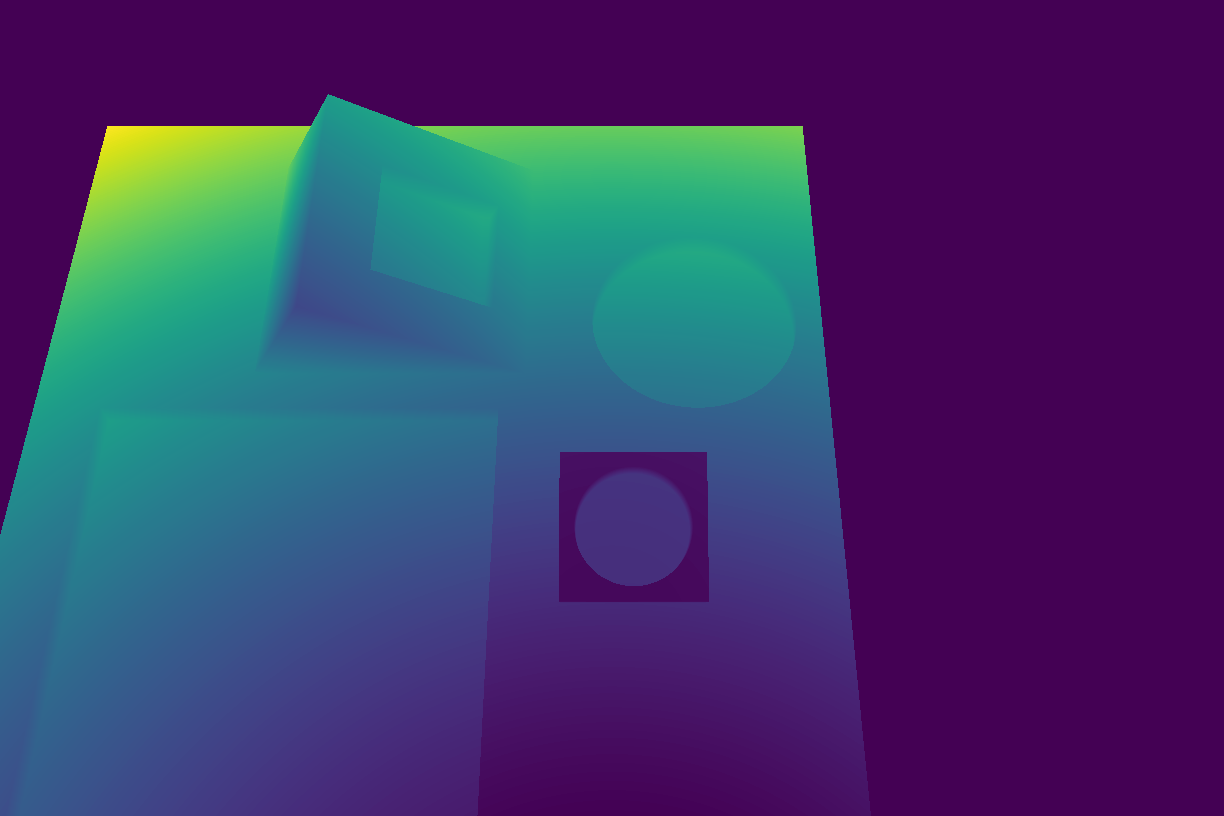

Supplement: Supplementary file 1 [file sensors-23-07964-s001.zip › Academic Example Synthetic/6_GC-Spaces/G1/render/vx7_depth_image_gcs_G1_s1.png]

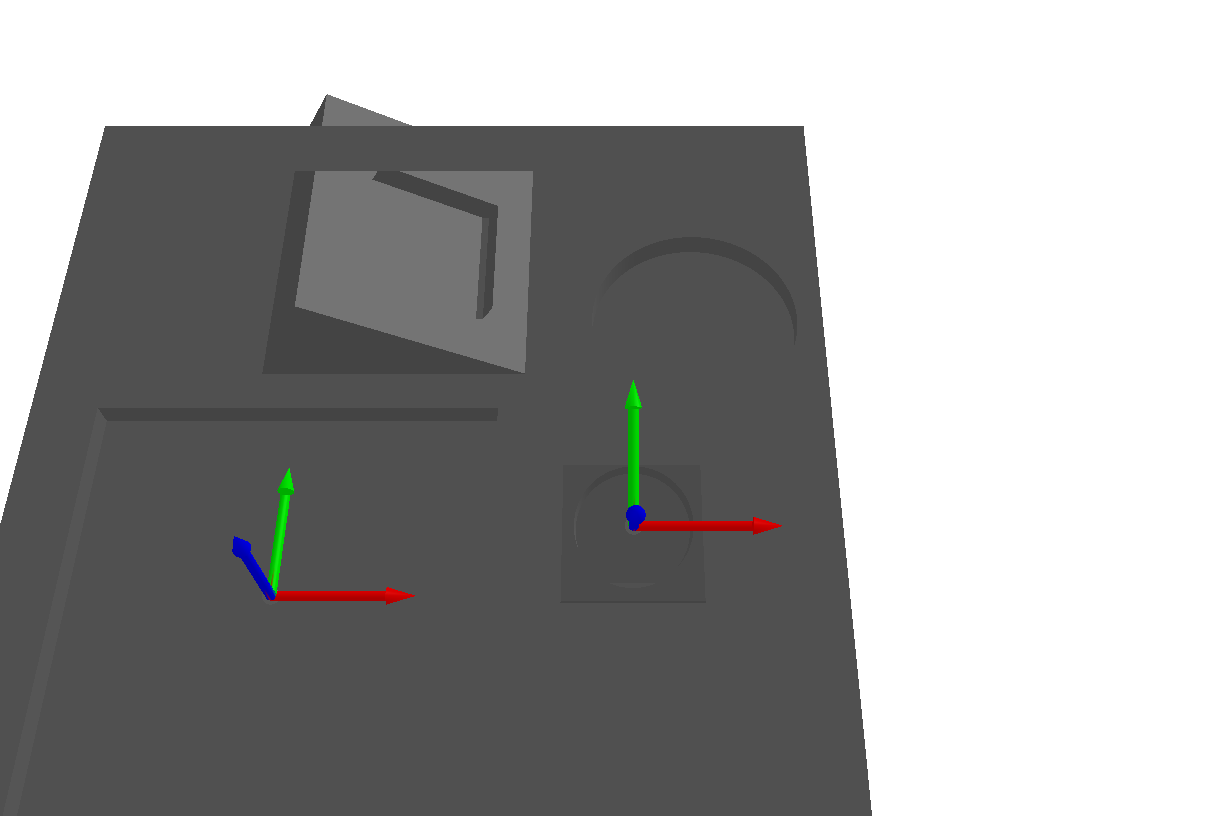

Supplement: Supplementary file 1 [file sensors-23-07964-s001.zip › Academic Example Synthetic/6_GC-Spaces/G1/render/vx7_render_gcs_G1_s1.png]

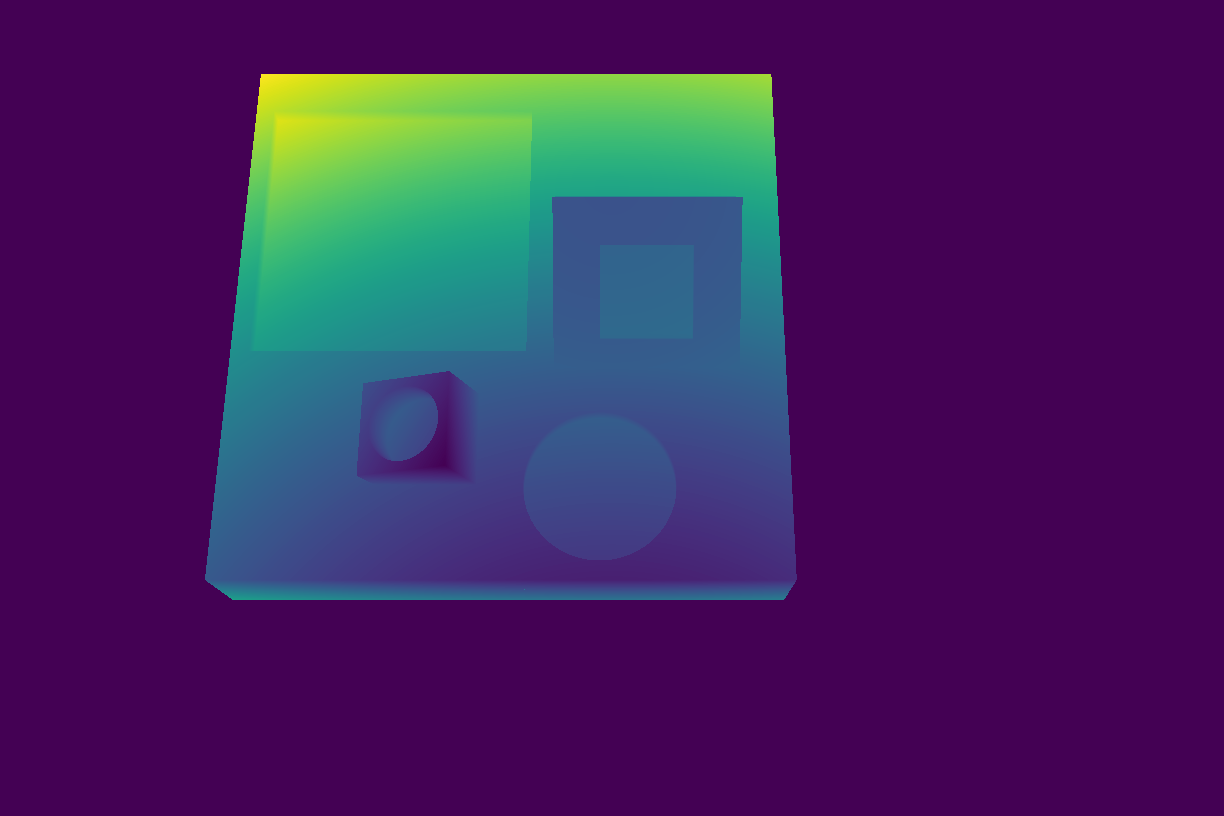

Supplement: Supplementary file 1 [file sensors-23-07964-s001.zip › Academic Example Synthetic/6_GC-Spaces/G2/render/center_depth_image_gcs_G2_s1.png]

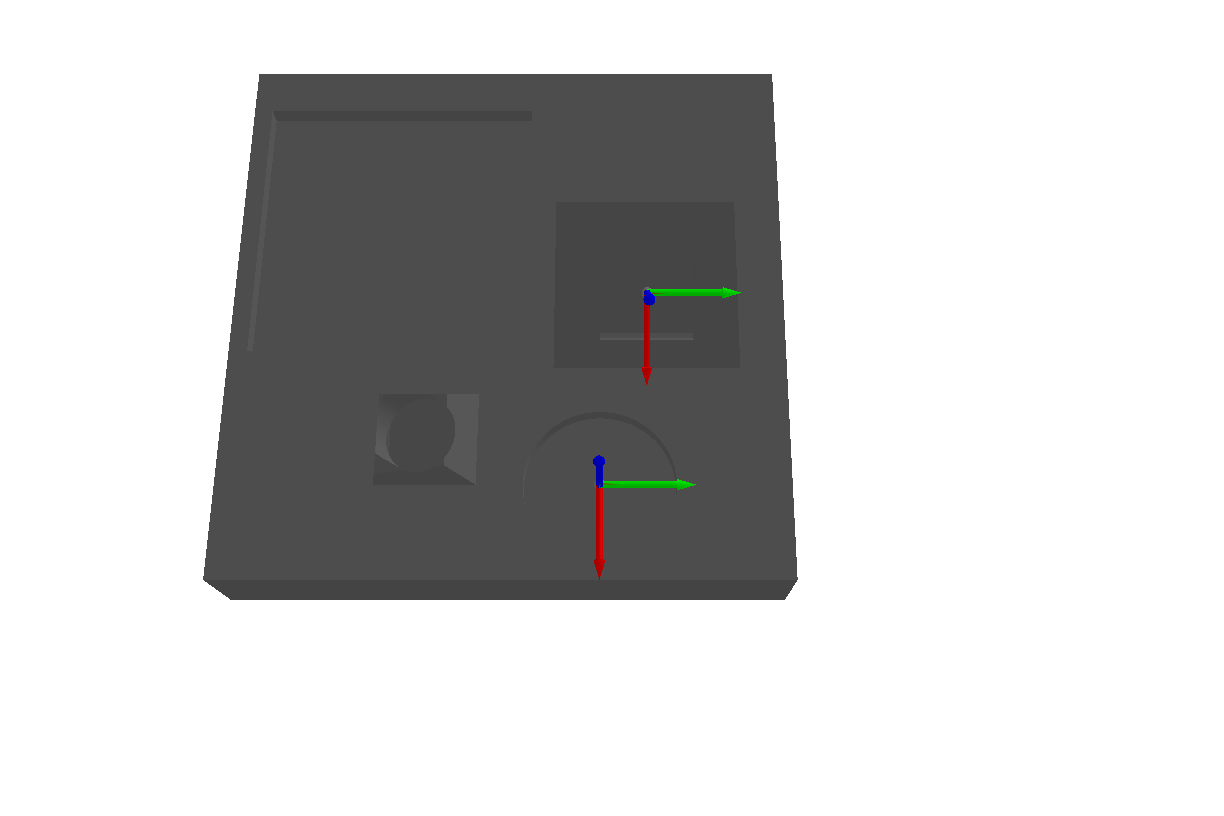

Supplement: Supplementary file 1 [file sensors-23-07964-s001.zip › Academic Example Synthetic/6_GC-Spaces/G2/render/center_render_gcs_G2_s1.png]

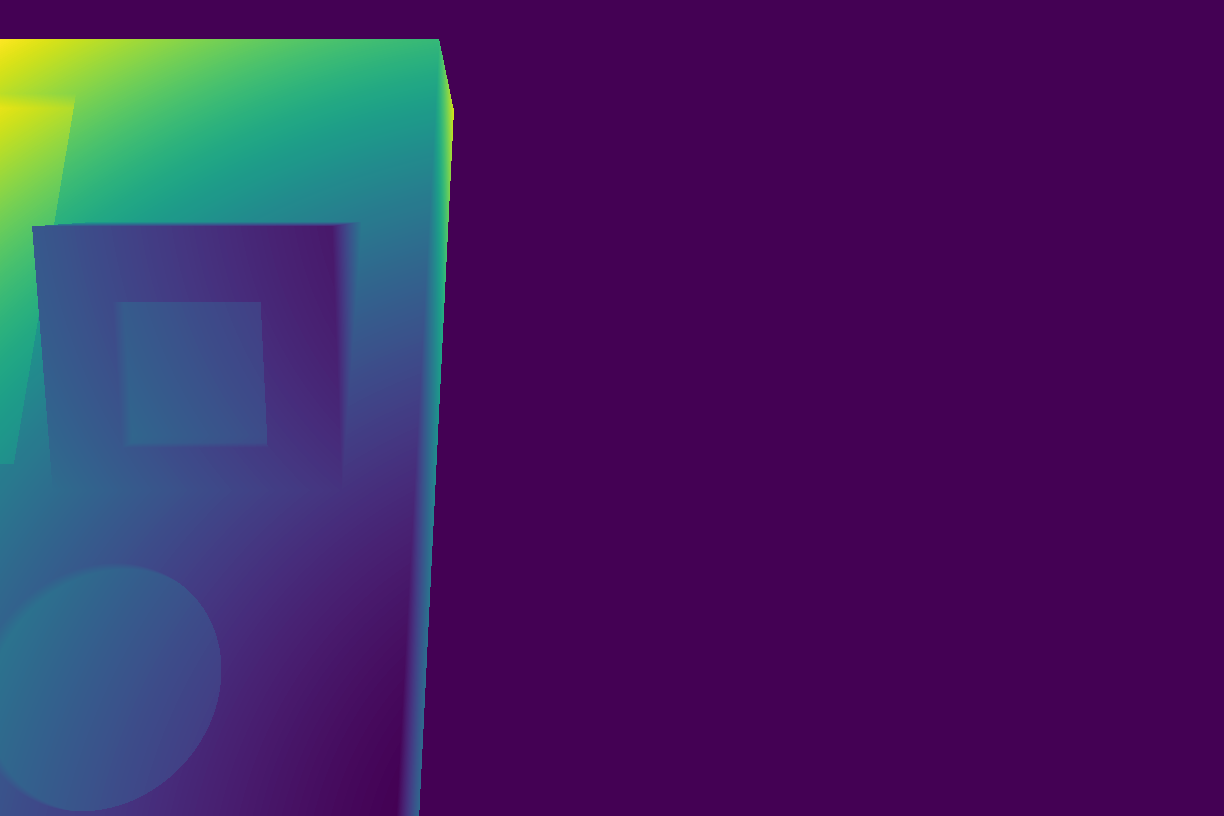

Supplement: Supplementary file 1 [file sensors-23-07964-s001.zip › Academic Example Synthetic/6_GC-Spaces/G2/render/vx0_depth_image_gcs_G2_s1.png]

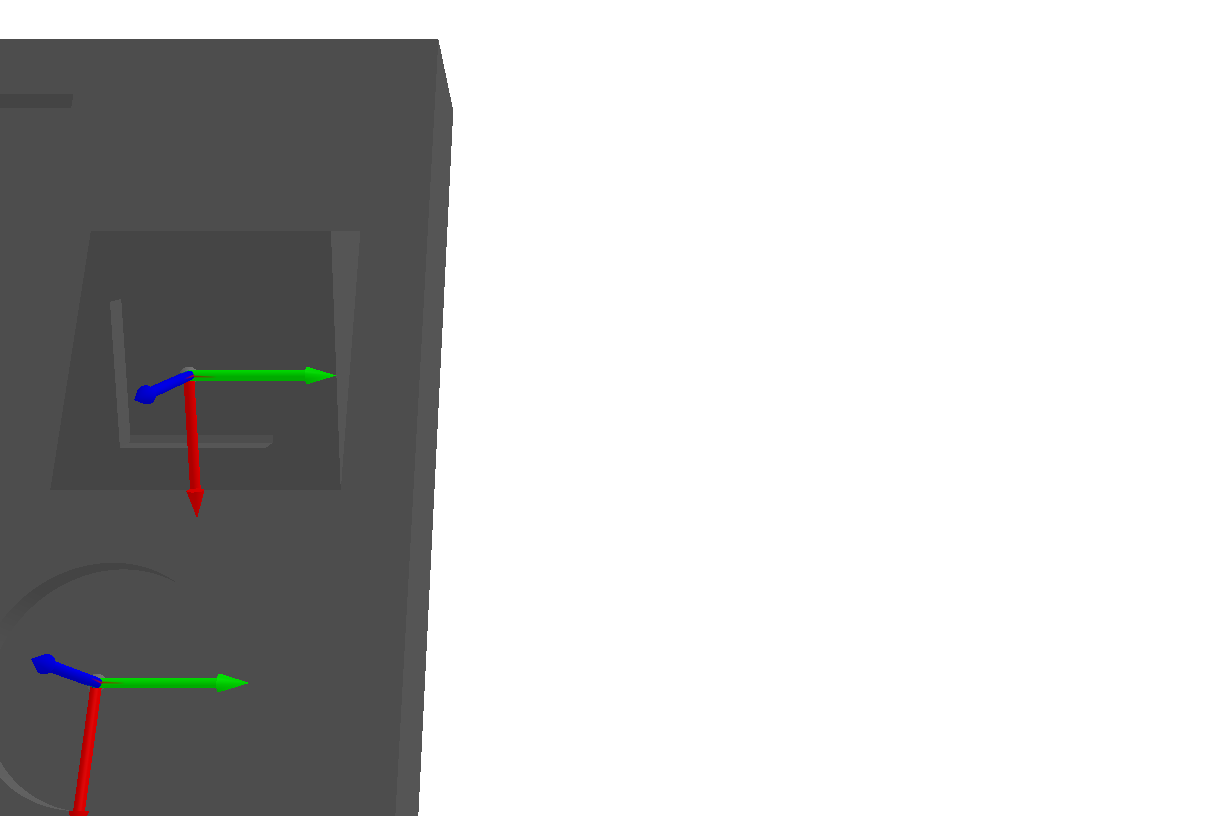

Supplement: Supplementary file 1 [file sensors-23-07964-s001.zip › Academic Example Synthetic/6_GC-Spaces/G2/render/vx0_render_gcs_G2_s1.png]

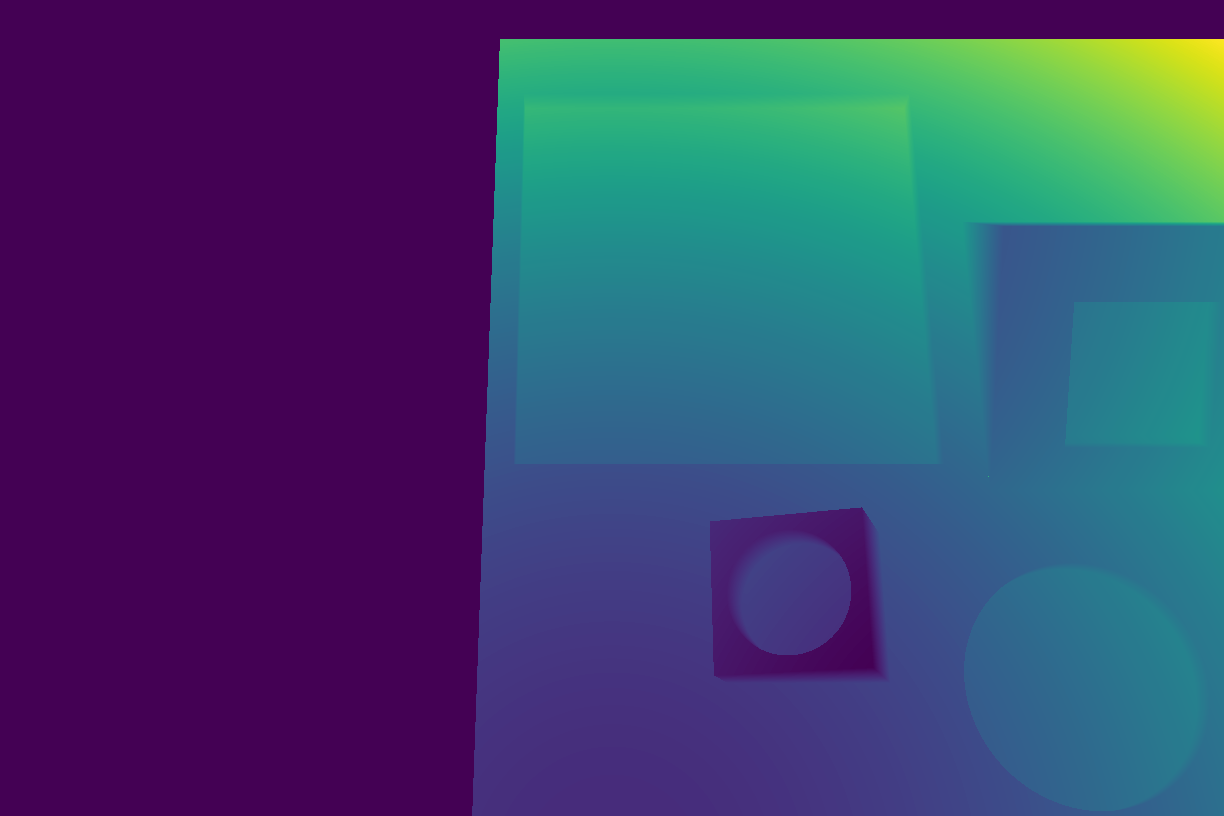

Supplement: Supplementary file 1 [file sensors-23-07964-s001.zip › Academic Example Synthetic/6_GC-Spaces/G2/render/vx1_depth_image_gcs_G2_s1.png]

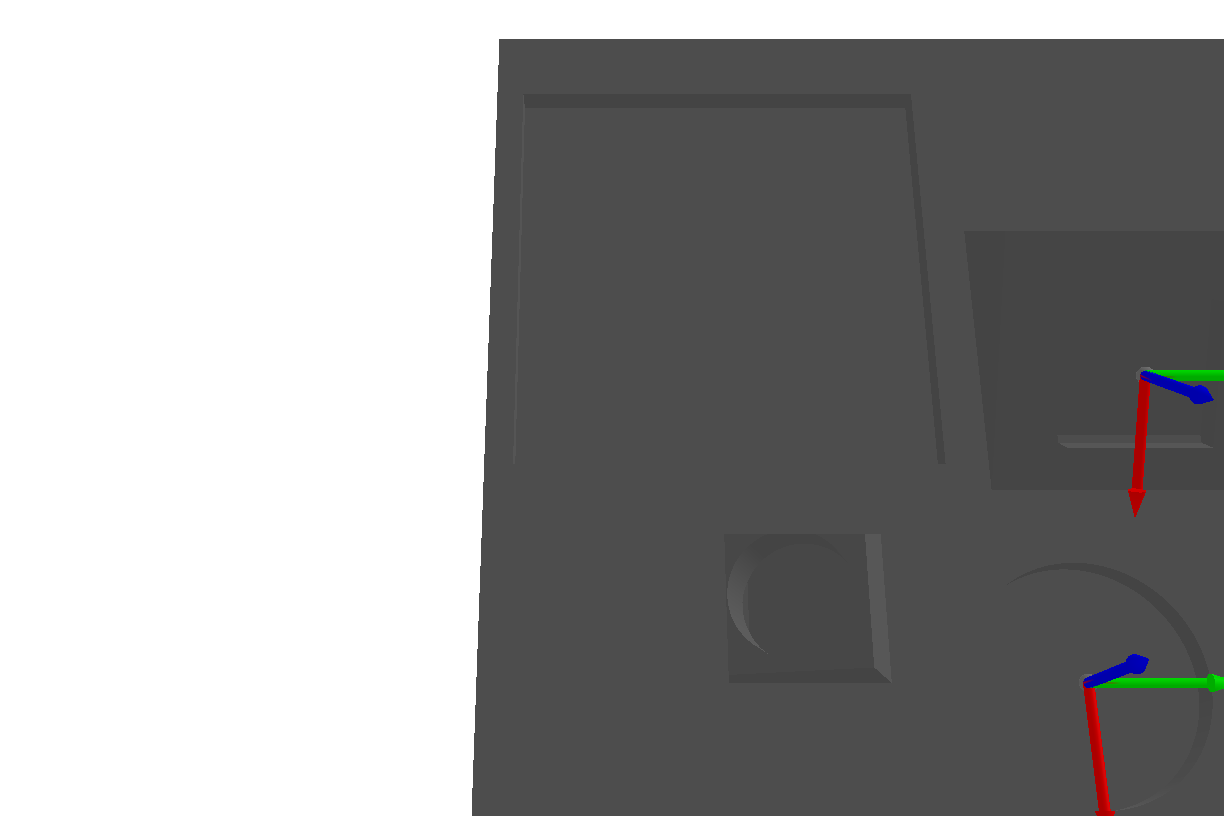

Supplement: Supplementary file 1 [file sensors-23-07964-s001.zip › Academic Example Synthetic/6_GC-Spaces/G2/render/vx1_render_gcs_G2_s1.png]

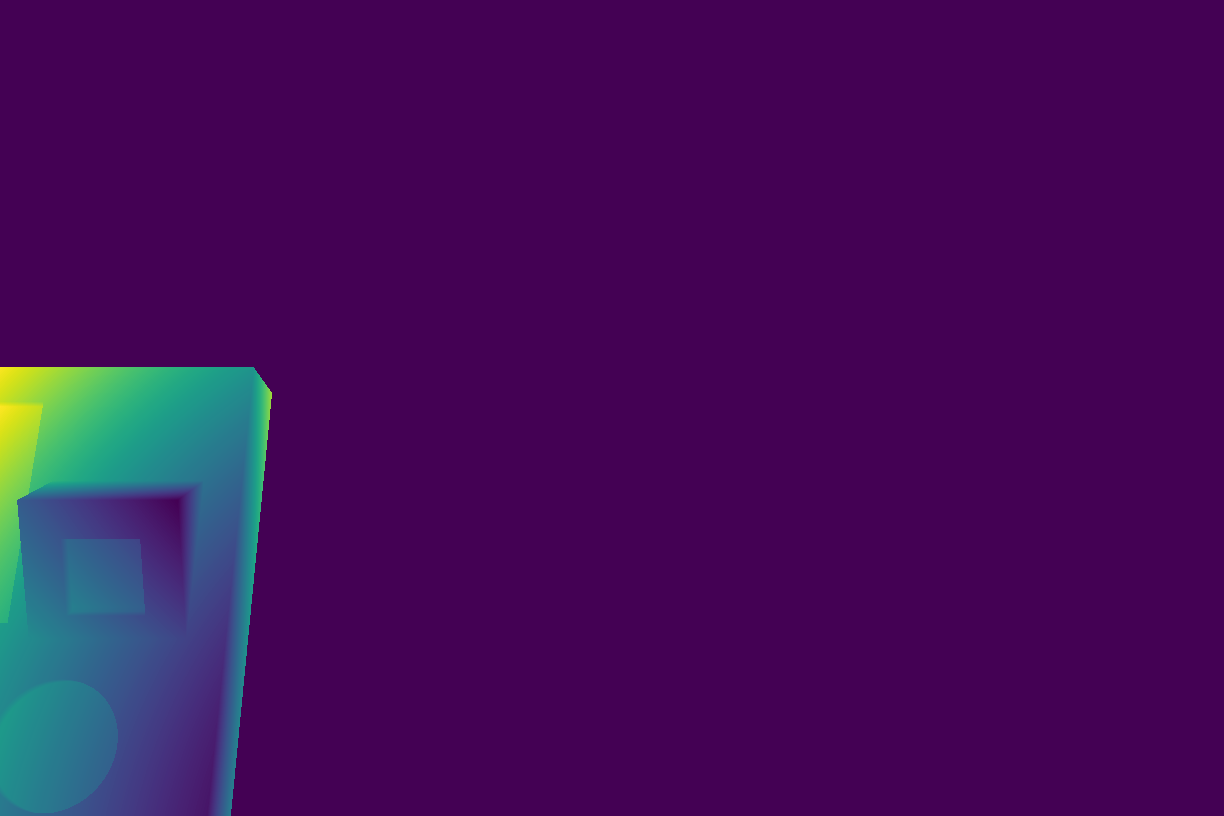

Supplement: Supplementary file 1 [file sensors-23-07964-s001.zip › Academic Example Synthetic/6_GC-Spaces/G2/render/vx2_depth_image_gcs_G2_s1.png]

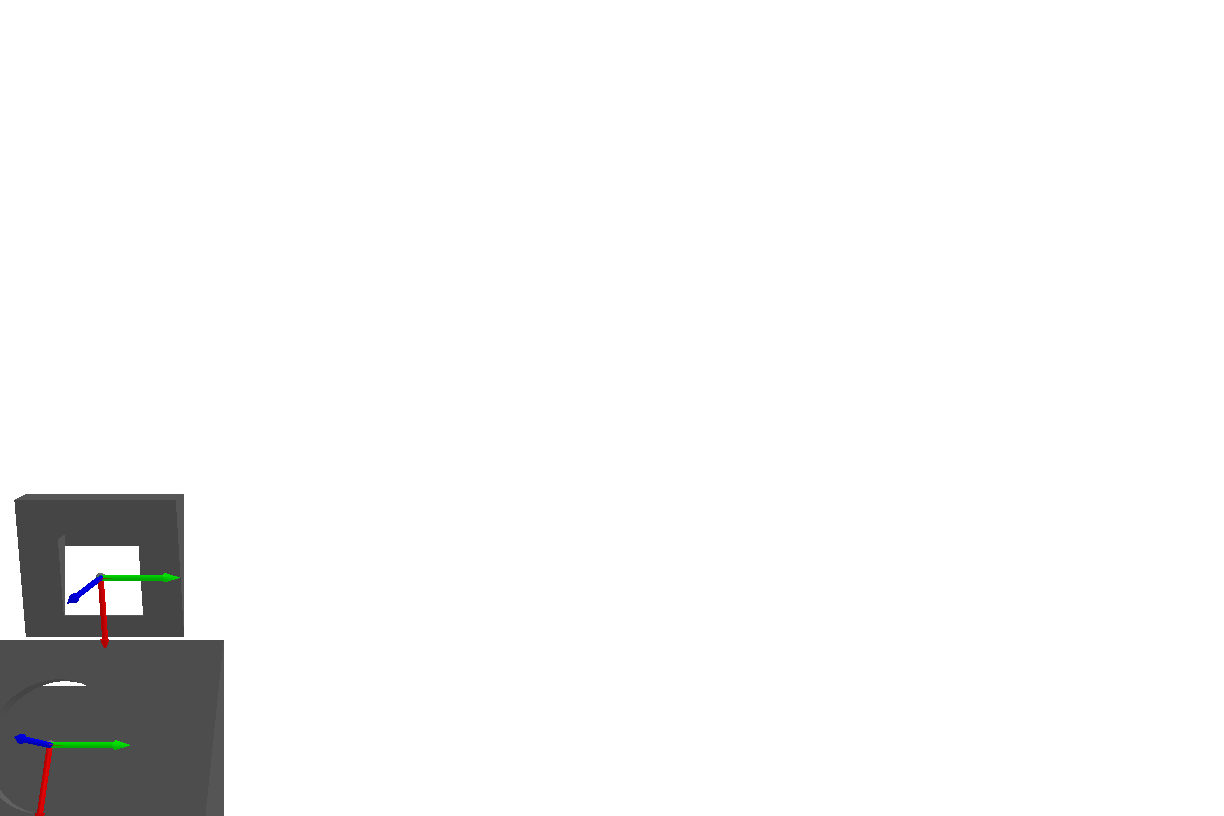

Supplement: Supplementary file 1 [file sensors-23-07964-s001.zip › Academic Example Synthetic/6_GC-Spaces/G2/render/vx2_render_gcs_G2_s1.png]

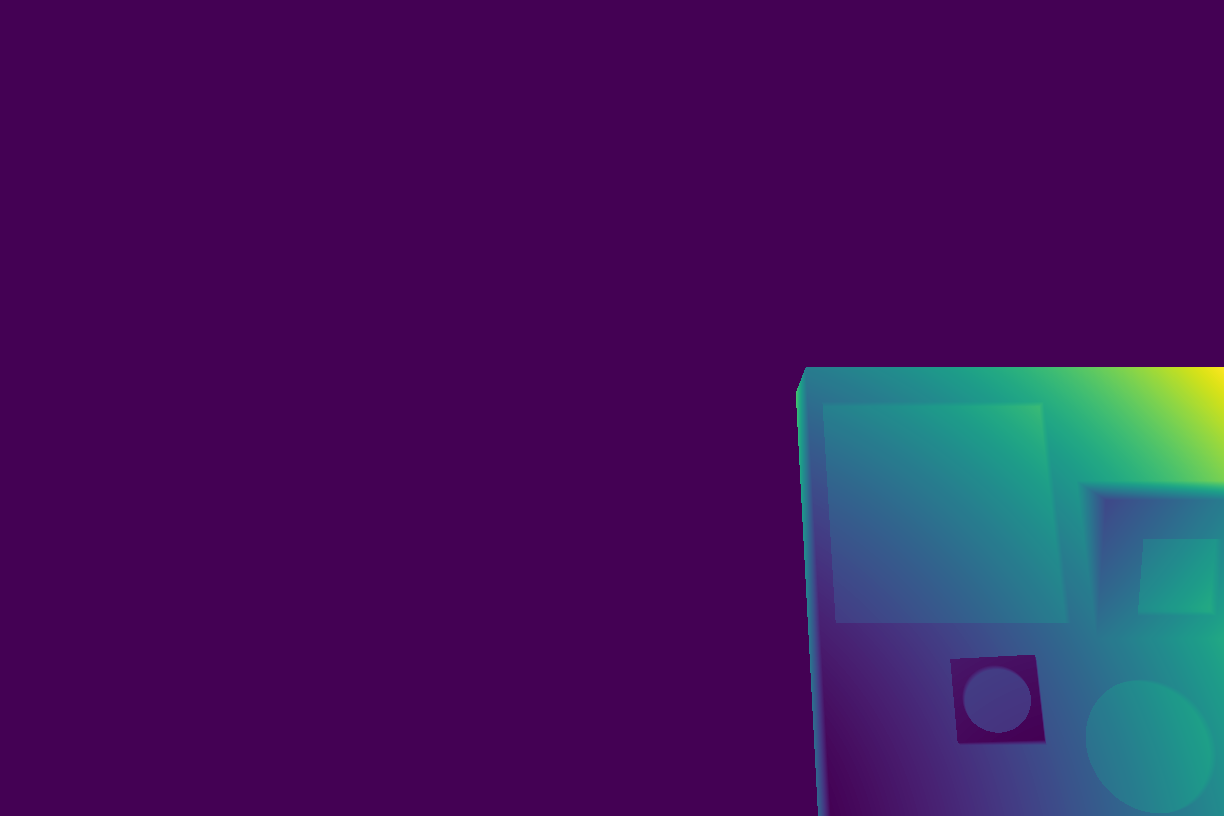

Supplement: Supplementary file 1 [file sensors-23-07964-s001.zip › Academic Example Synthetic/6_GC-Spaces/G2/render/vx3_depth_image_gcs_G2_s1.png]

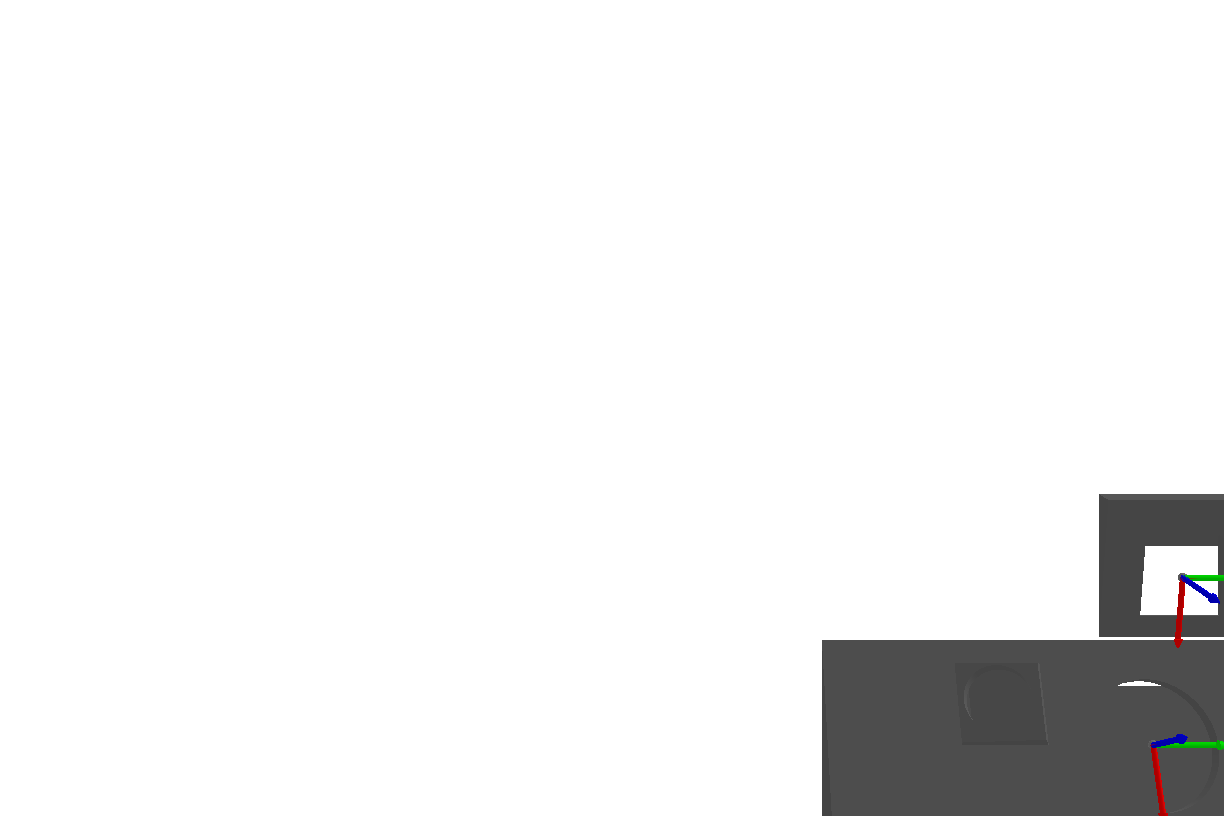

Supplement: Supplementary file 1 [file sensors-23-07964-s001.zip › Academic Example Synthetic/6_GC-Spaces/G2/render/vx3_render_gcs_G2_s1.png]

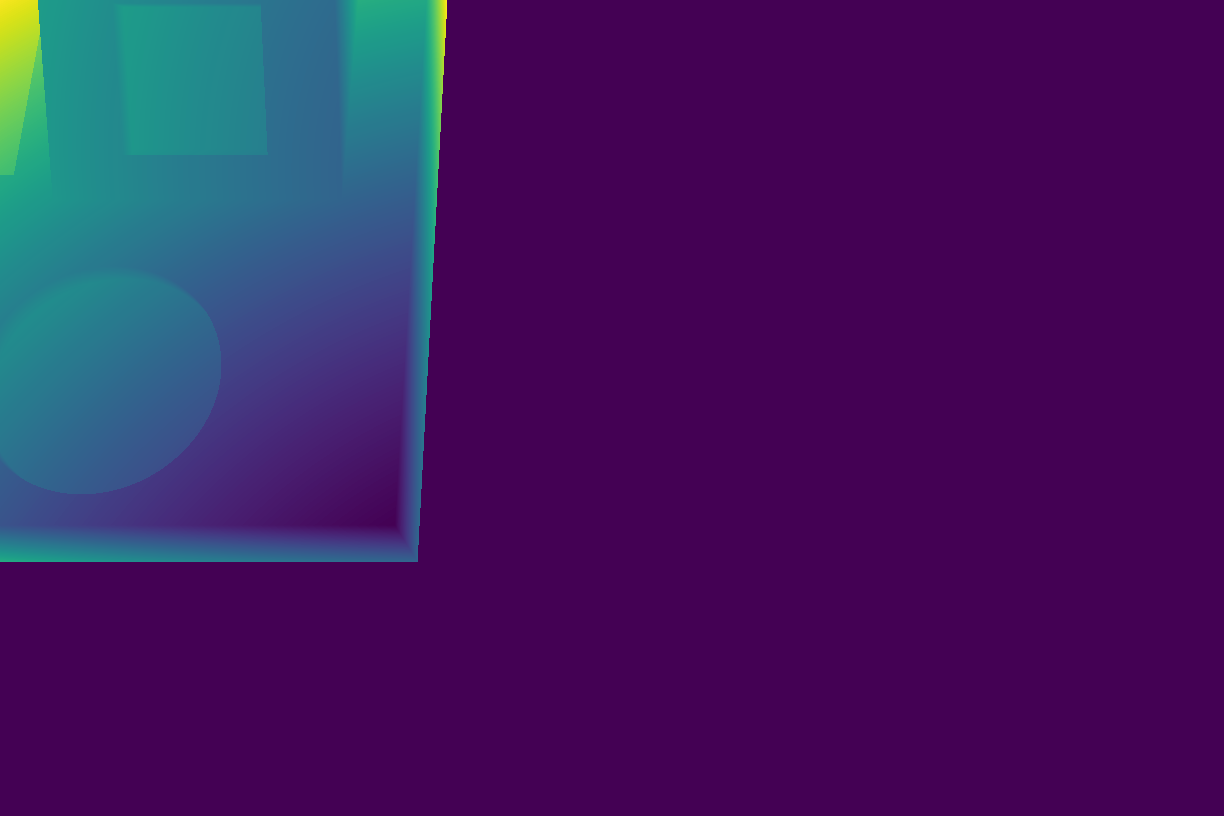

Supplement: Supplementary file 1 [file sensors-23-07964-s001.zip › Academic Example Synthetic/6_GC-Spaces/G2/render/vx4_depth_image_gcs_G2_s1.png]

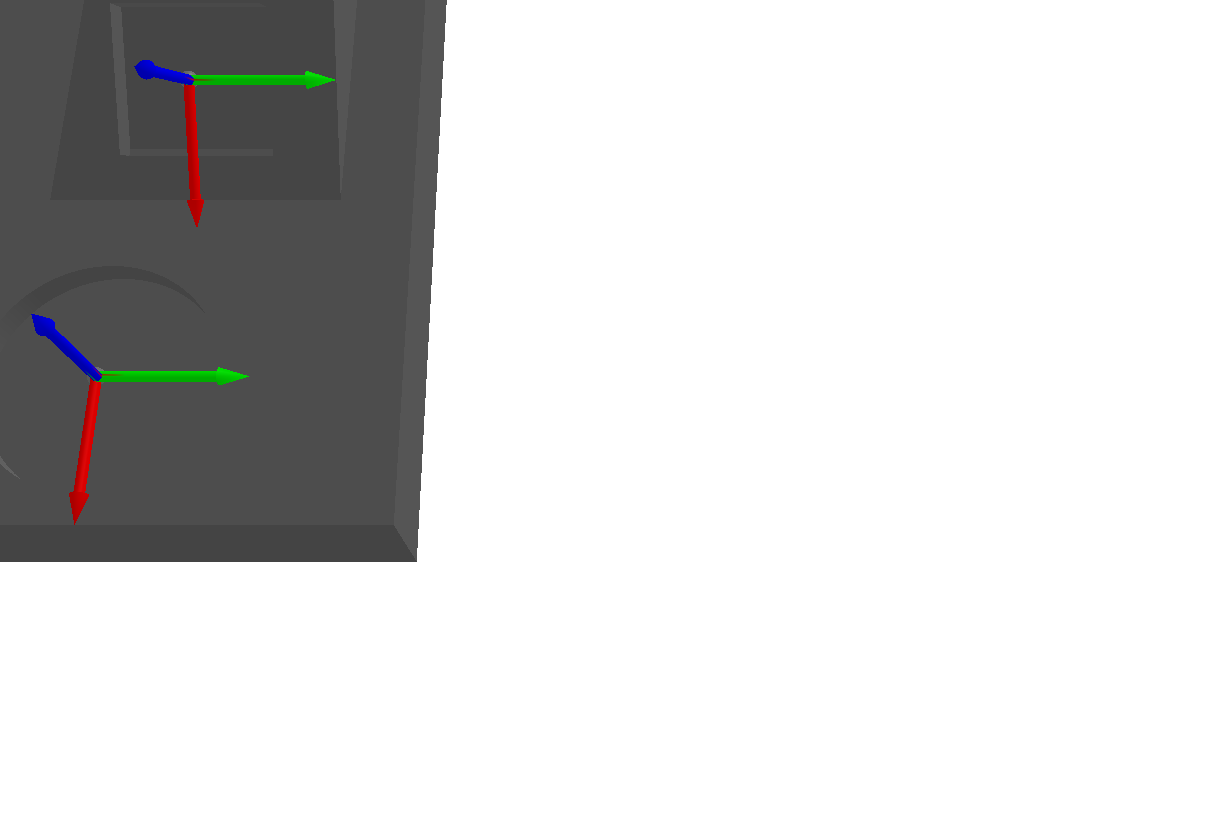

Supplement: Supplementary file 1 [file sensors-23-07964-s001.zip › Academic Example Synthetic/6_GC-Spaces/G2/render/vx4_render_gcs_G2_s1.png]

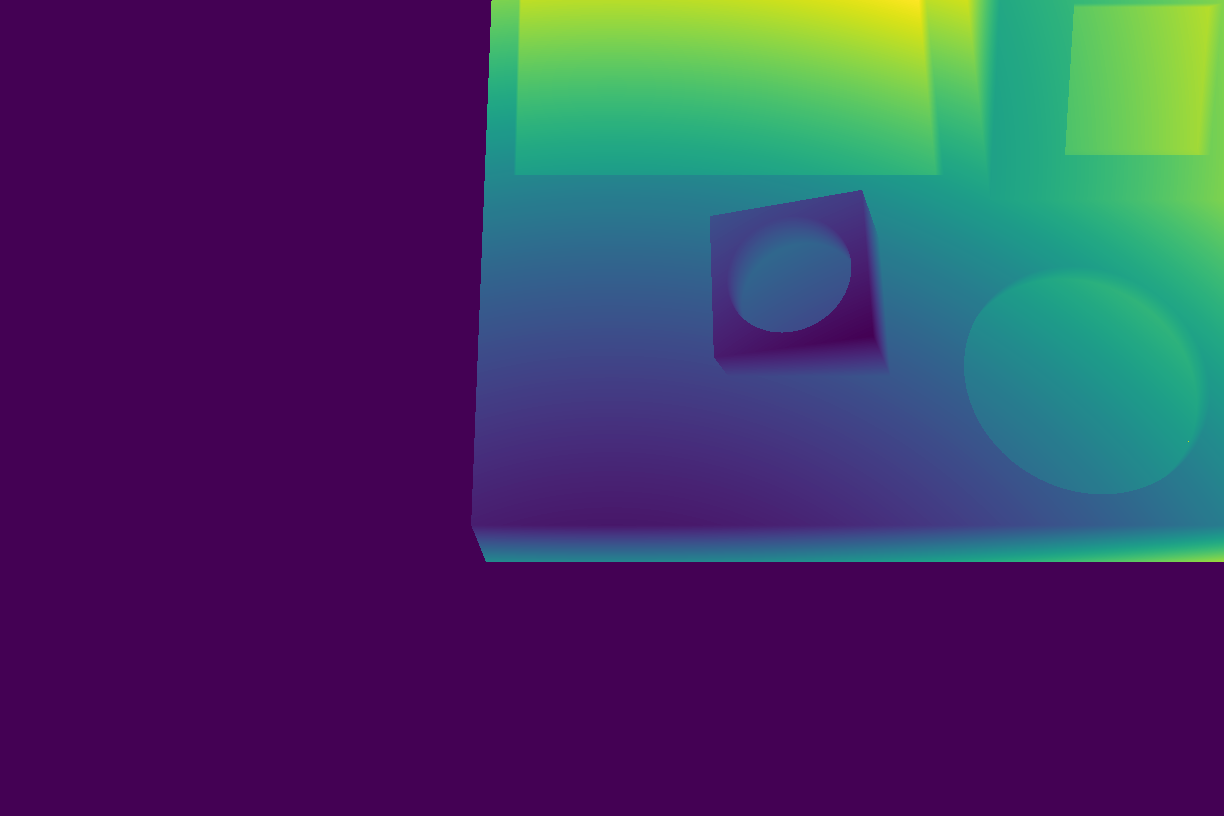

Supplement: Supplementary file 1 [file sensors-23-07964-s001.zip › Academic Example Synthetic/6_GC-Spaces/G2/render/vx5_depth_image_gcs_G2_s1.png]

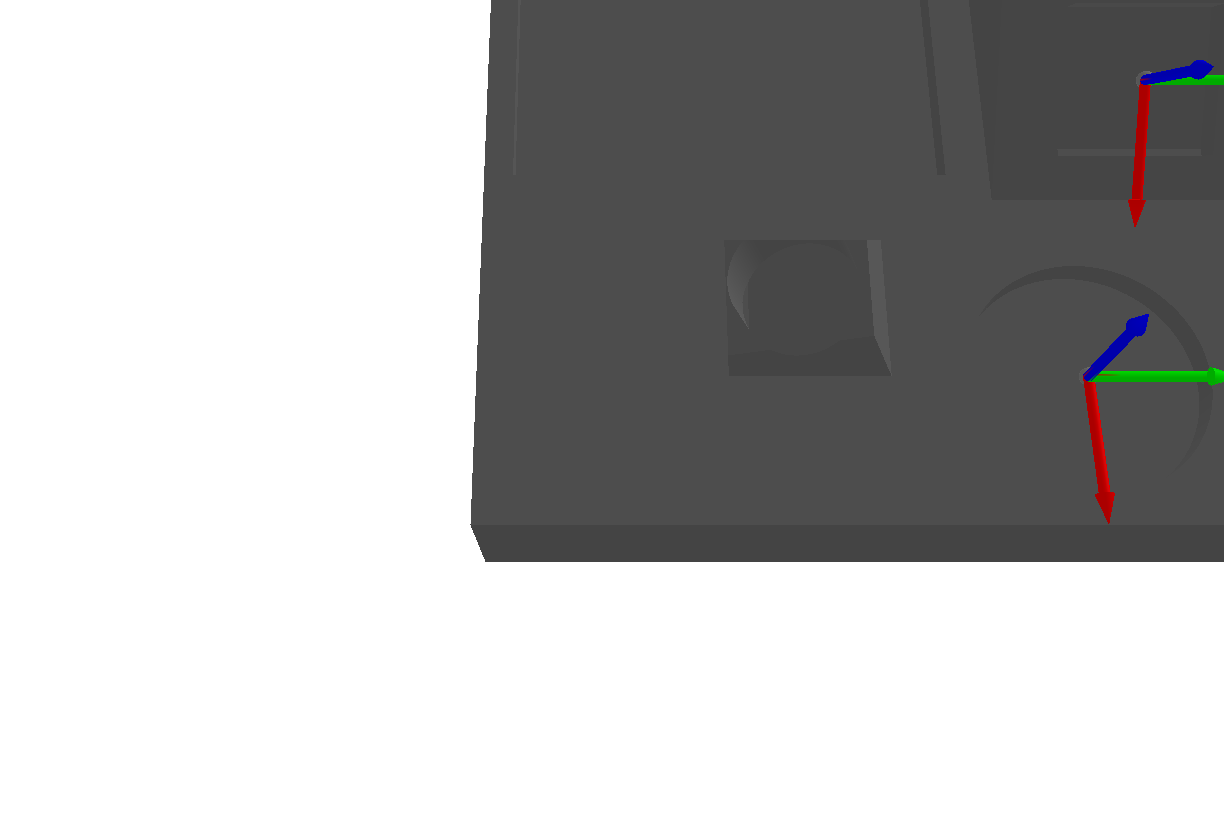

Supplement: Supplementary file 1 [file sensors-23-07964-s001.zip › Academic Example Synthetic/6_GC-Spaces/G2/render/vx5_render_gcs_G2_s1.png]

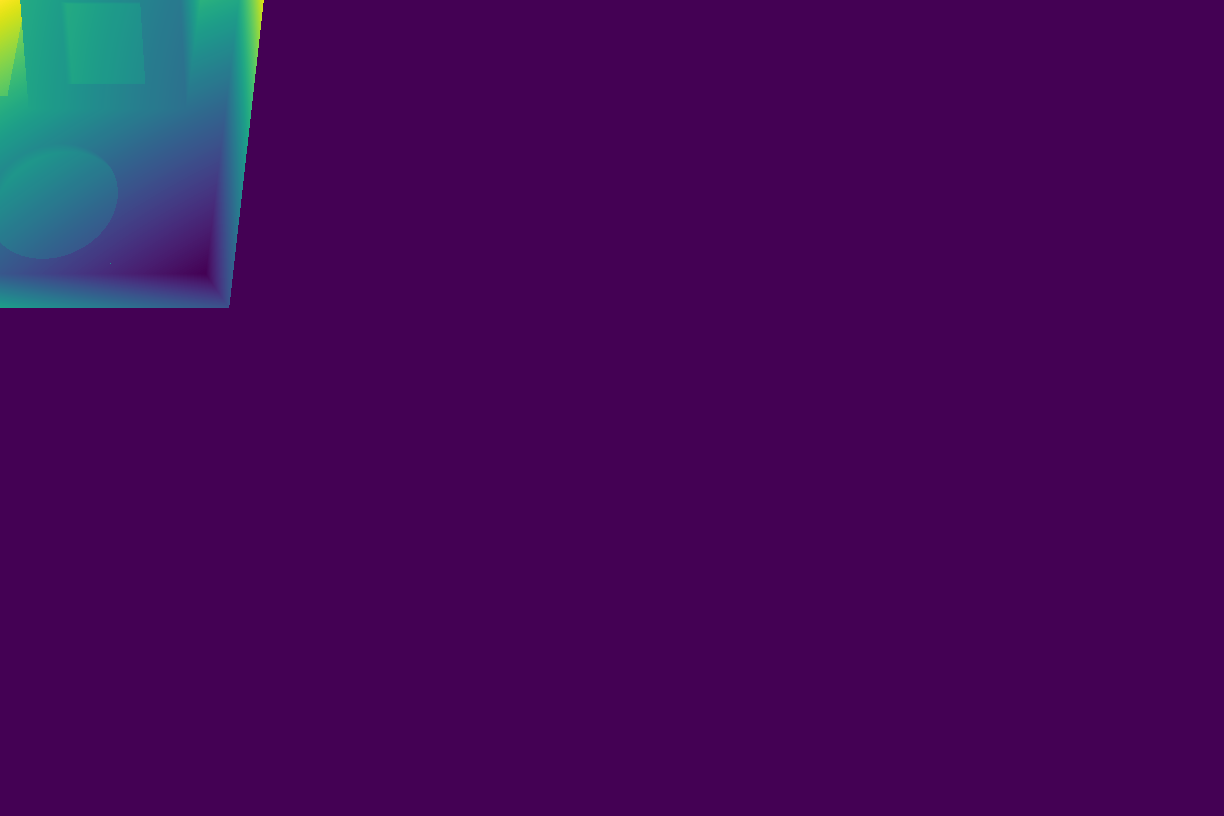

Supplement: Supplementary file 1 [file sensors-23-07964-s001.zip › Academic Example Synthetic/6_GC-Spaces/G2/render/vx6_depth_image_gcs_G2_s1.png]

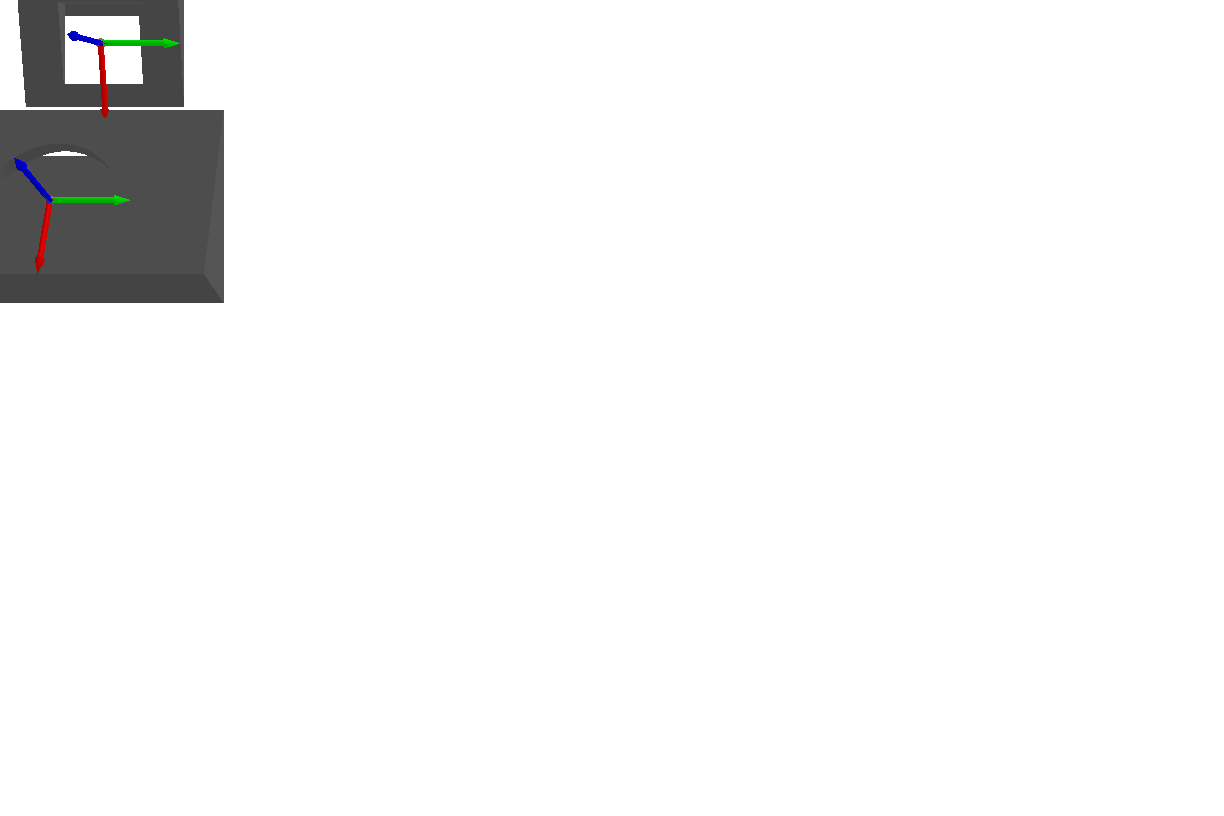

Supplement: Supplementary file 1 [file sensors-23-07964-s001.zip › Academic Example Synthetic/6_GC-Spaces/G2/render/vx6_render_gcs_G2_s1.png]

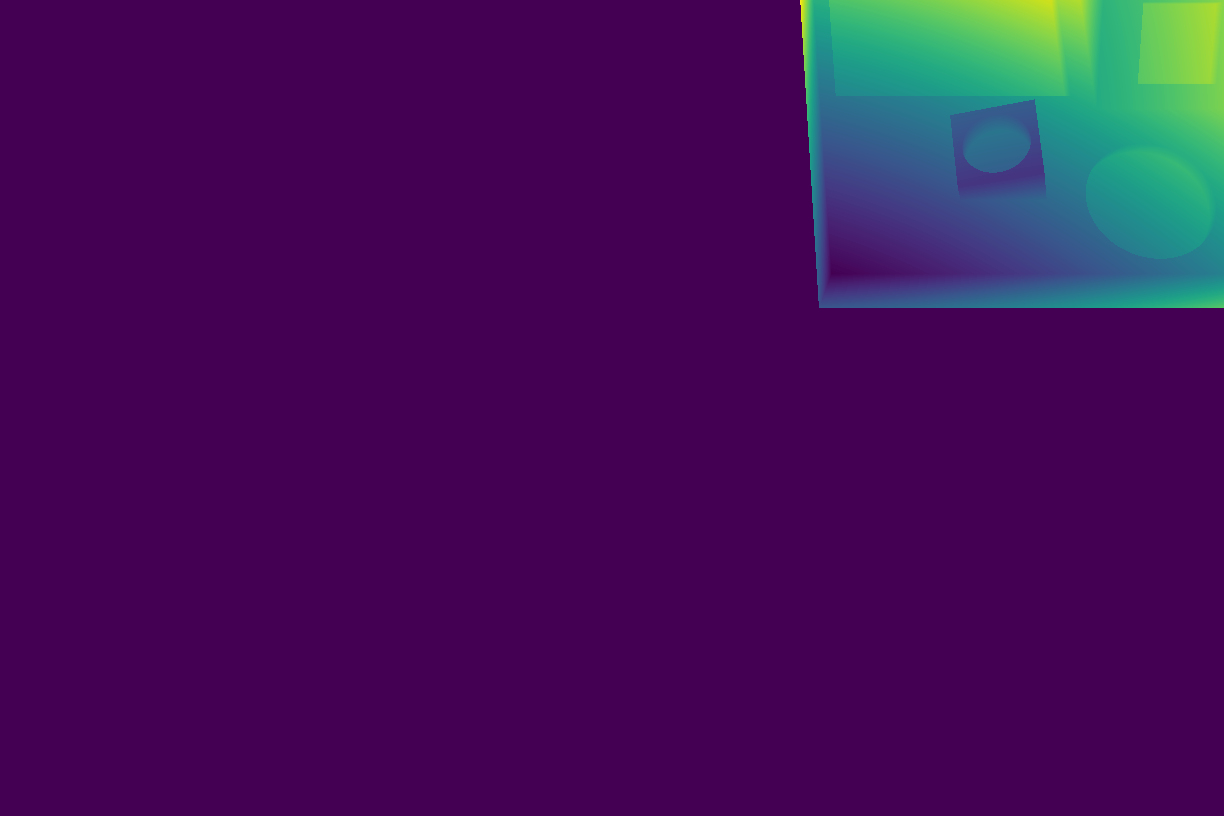

Supplement: Supplementary file 1 [file sensors-23-07964-s001.zip › Academic Example Synthetic/6_GC-Spaces/G2/render/vx7_depth_image_gcs_G2_s1.png]

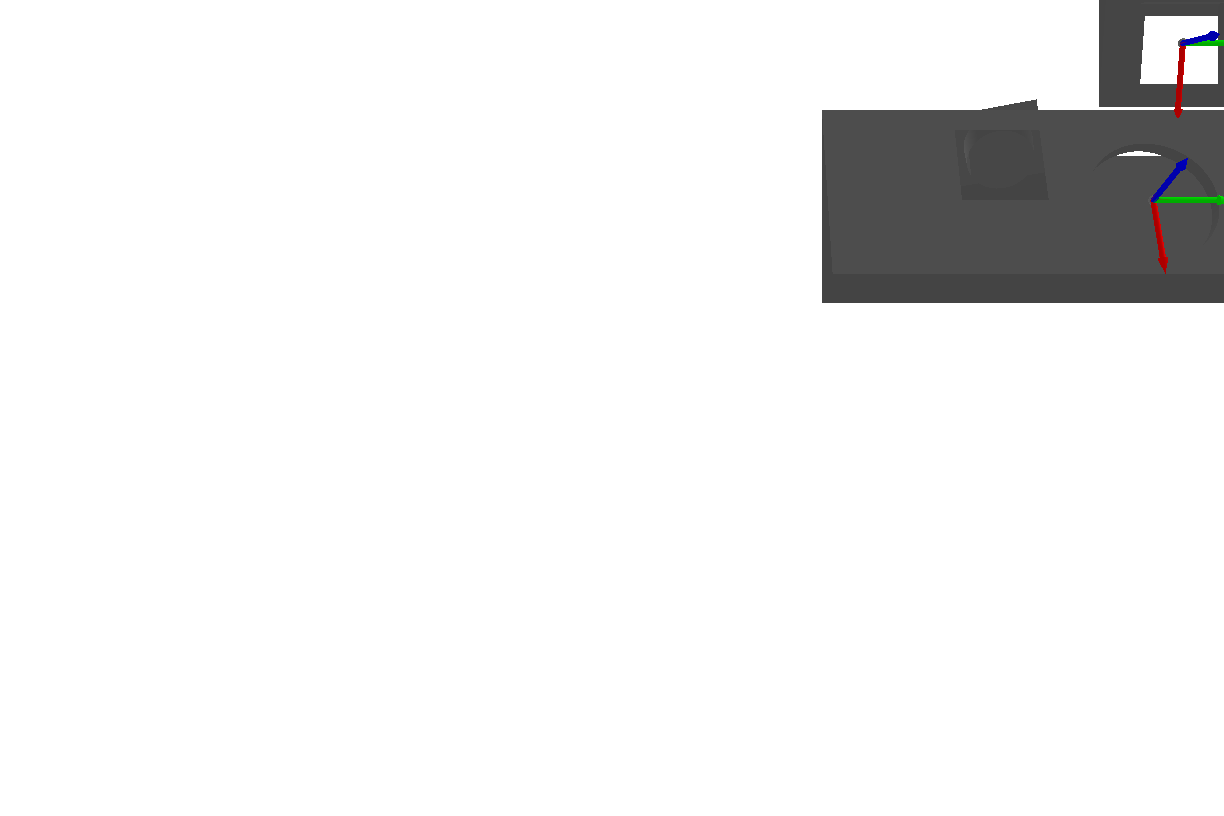

Supplement: Supplementary file 1 [file sensors-23-07964-s001.zip › Academic Example Synthetic/6_GC-Spaces/G2/render/vx7_render_gcs_G2_s1.png]

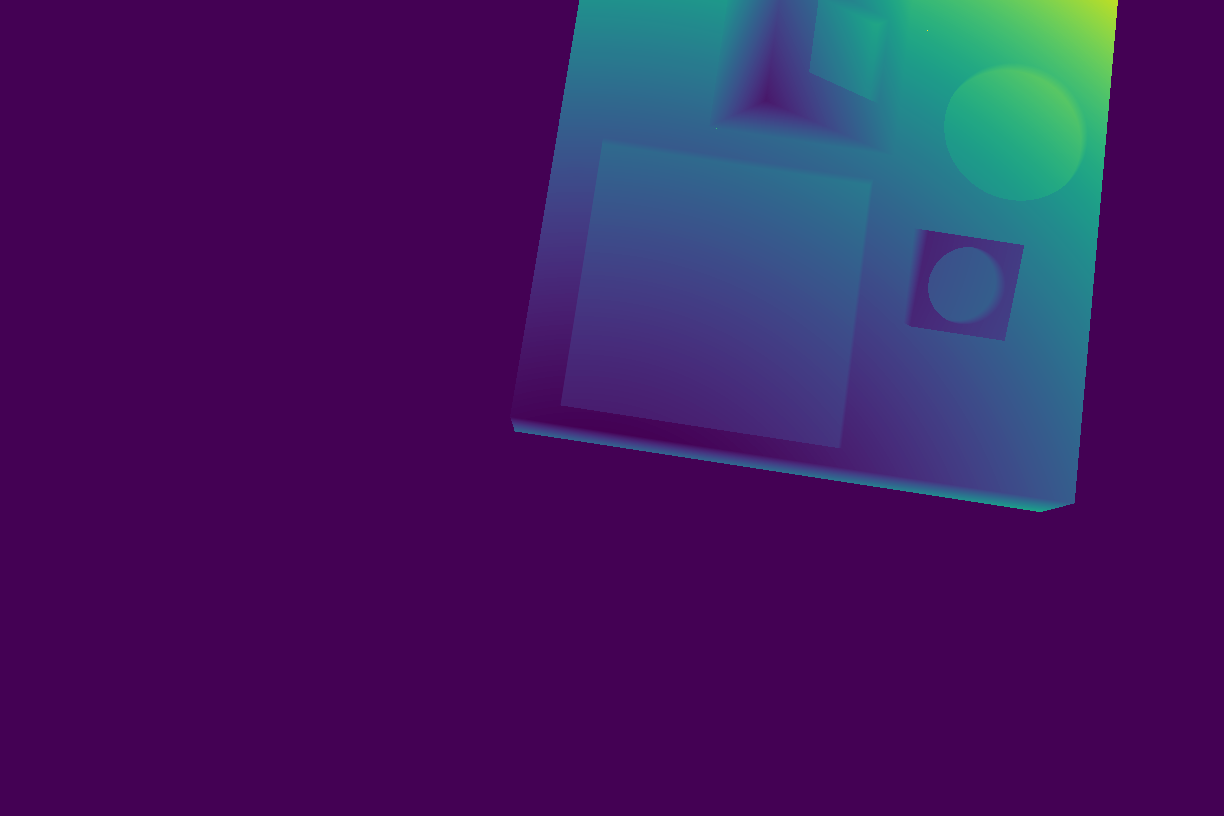

Supplement: Supplementary file 1 [file sensors-23-07964-s001.zip › Academic Example Synthetic/7_Strategy/G1/render/center_depth_image_gcs_G1_s1.png]

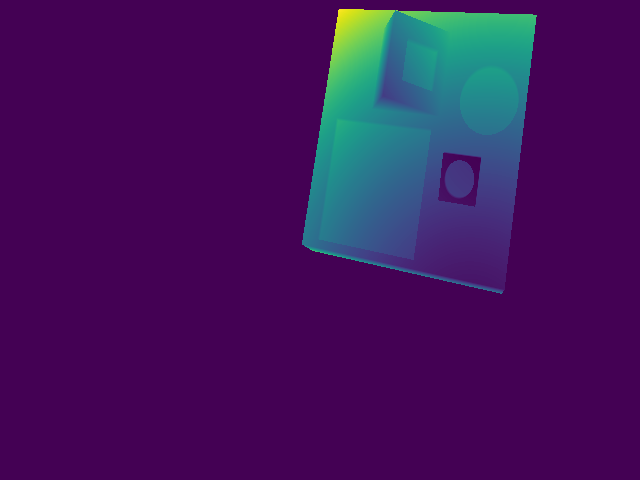

Supplement: Supplementary file 1 [file sensors-23-07964-s001.zip › Academic Example Synthetic/7_Strategy/G1/render/center_depth_image_gcs_G1_s2.png]
